# Vedolizumab for the prevention of intestinal acute GVHD after allogeneic hematopoietic stem cell transplantation: a randomized phase 3 trial

**DOI:** 10.1038/s41591-024-03016-4

**Published:** 2024-06-06

**Authors:** Yi-Bin Chen, Mohamad Mohty, Robert Zeiser, Takanori Teshima, Omer Jamy, Johan Maertens, Duncan Purtill, Jingjing Chen, Hong Cao, Guillermo Rossiter, Johan Jansson, Yngvar Fløisand

**Affiliations:** 1 Hematopoietic Cell Transplant and Cellular Therapy Program, Massachusetts General Hospital, Boston, MA USA; 2 Hematology Department, AP-HP, Hôpital Saint-Antoine, Sorbonne Université and INSERM UMRs 938, Paris, France; 3 Department of Medicine I - Medical Centre, Faculty of Medicine, University of Freiburg, Freiburg, Germany; 4 Department of Hematology, Hokkaido University Faculty of Medicine, Sapporo, Japan; 5 Division of Hematology and Oncology, University of Alabama at Birmingham, Birmingham, AL USA; 6 Department of Hematology, University Hospitals Leuven, Leuven, Belgium; 7 Department of Microbiology, Immunology, and Transplantation, KU Leuven, Leuven, Belgium; 8 Department of Haematology, Fiona Stanley Hospital, Perth, Western Australia Australia; 9 PathWest Laboratory Medicine, Perth, Western Australia Australia; 10 Takeda, Cambridge, MA USA; 11 Centre for Cancer Cell Reprogramming, Institute of Clinical Medicine, Faculty of Medicine, University of Oslo, Oslo, Norway

**Keywords:** Randomized controlled trials, Graft-versus-host disease

## Abstract

Acute graft-versus-host disease (aGVHD) of the lower gastrointestinal (GI) tract is a major cause of morbidity and mortality in patients receiving allogeneic hematopoietic stem cell transplantation (allo-HSCT). Vedolizumab is a gut-selective anti-α_4_β_7_ integrin monoclonal antibody that reduces gut inflammation by inhibiting migration of GI-homing T lymphocytes. The efficacy and safety of vedolizumab added to standard GVHD prophylaxis (calcineurin inhibitor plus methotrexate/mycophenolate mofetil) was evaluated for prevention of lower-GI aGVHD after unrelated donor allo-HSCT in a randomized, double-blind, placebo-controlled phase 3 trial. Enrollment closed early during the COVID-19 pandemic with 343 patients randomized (*n* = 174 vedolizumab, *n* = 169 placebo), and 333 received ≥1 intravenous dose of 300 mg vedolizumab (*n* = 168) or placebo (*n* = 165) and underwent allo-HSCT. The primary end point was met; Kaplan–Meier (95% confidence interval) estimated rates of lower-GI aGVHD-free survival by day +180 after allo-HSCT were 85.5% (79.2–90.1) with vedolizumab versus 70.9% (63.2–77.2) with placebo (hazard ratio, 0.45; 95% confidence interval, 0.27–0.73; *P* < 0.001). For the 5 key secondary efficacy end points analyzed by day +180 after allo-HSCT, rates of lower-GI aGVHD-free and relapse-free survival and grade C–D aGVHD-free survival were significantly higher with vedolizumab versus placebo. No significant treatment differences were found for the other key secondary end points of non-relapse mortality, overall survival and grade B–D aGVHD-free survival, respectively. Incidence of treatment-related serious adverse events analyzed in patients receiving ≥1 dose of study treatment (*n* = 334) was 6.5% (*n* = 11 of 169) vedolizumab versus 8.5% (*n* = 14 of 165) placebo. When added to standard calcineurin inhibitor-based GVHD prevention, lower-GI aGVHD-free survival was significantly higher with vedolizumab versus placebo. ClinicalTrials.gov identifier: NCT03657160.

## Abstract (web summary)

In a randomized, double-blind phase 3 trial, the anti-α_4_β_7_ integrin monoclonal antibody vedolizumab plus standard prophylaxis was superior to placebo plus prophylaxis for prevention of lower gastrointestinal acute graft-versus-host disease in patients following allogeneic hematopoietic stem cell transplantation.

## Main

Acute graft-versus-host disease (aGVHD) remains a major cause of morbidity and mortality after allogeneic hematopoietic stem cell transplantation (allo-HSCT) for hematologic malignancies^1–4^. GVHD results from the activation of donor T cells migrating to target organs and exerting their effector functions^5–7^. aGVHD commonly affects the skin, gastrointestinal (GI) tract and liver^3,8^. aGVHD is classified according to the extent and severity of individual organ involvement (stages 0–4) on the basis of objective clinical criteria^9^. The stages are combined to generate an overall clinical grade (grades 0–4 in the modified Glucksberg system, Mount Sinai Acute GVHD International Consortium (MAGIC) system and grades A–D in the International Bone Marrow Transplant Registry Database (IBMTR) system; Supplementary Tables 1–4)^9–11^. Grade 2–4 aGVHD develops in ~40–50% of well-matched allo-HSCT recipients, despite standard GVHD prophylaxis^12,13^. Multiple factors influence aGVHD risk, including stem cell source, patient and donor age, conditioning regimen intensity and choice of GVHD prophylaxis. The majority of the morbidity and mortality associated with aGVHD is driven by aGVHD of the lower GI tract^14,15^; this is initiated by local release of damage-associated molecular patterns and microbial components that penetrate the damaged intestinal wall^14,16^. The overall incidence of lower-GI stage 2–4 aGVHD is ~15–26% in allo-HSCT recipients receiving standard calcineurin inhibitor (CNI)-based prophylaxis^15,17,18^.

The pathophysiology of aGVHD involves the stimulation of allogenic donor T cells by recipient allo-antigens and the migration of T cells, macrophages and neutrophils to the intestinal wall^19^. Studies using murine models of allogeneic bone marrow transplantation have established that α_4_β_7_ integrin receptor-mediated trafficking of T cells to gut lymphoid tissue has an important role in the gut invasion of alloreactive donor T cells, leading to the development of aGVHD^20,21^. Further analysis using three-dimensional (3D) microscopy to visualize trafficking of allogeneic T cells in the GI tract of a mouse GVHD model, found that donor-derived alloreactive T cells were primarily targeted to the intestinal stem cell compartment during immune-mediated tissue damage after transplant. This evidence suggested that early T cell infiltration could be blocked by disrupting the interaction of α_4_β_7_ integrin with its adhesion molecule ligand MAdCAM-1 found clustered on vasculature in the same area^22^.

Vedolizumab is a humanized monoclonal antibody that specifically binds to the α_4_β_7_ integrin leukocyte receptor^23^ and blocks its interaction with MAdCAM-1 on the endothelial cells of GI lymphoid tissue. This binding prevents α_4_β_7_-expressing T cells from entering gut tissue, which reduces inflammation of the GI tract^23–25^. Vedolizumab has established efficacy as a treatment for inflammatory bowel diseases such as Crohn’s disease, ulcerative colitis and pouchitis^26–29^. Its mechanism of action, disrupting the homing of alloreactive T cells and transmigration to GI lymphoid tissue, is also likely to be effective for prevention of intestinal aGVHD^30^. Moreover, expression of α_4_β_7_ integrin on the surface of naive and memory T cells was demonstrated to be upregulated in patients who developed intestinal aGVHD after allo-HSCT^31,32^. Previously, vedolizumab was well tolerated when added to standard CNI-based GVHD prophylaxis in adults undergoing allo-HSCT in a phase 1b clinical study^33^.

Here we report results from an international double-blind, randomized, placebo-controlled phase 3 trial to evaluate vedolizumab added to standard GVHD prophylaxis after unrelated allo-HSCT.

## Results

### Patients

Patients eligible for the study were aged ≥12 years, weighed ≥30 kg and had an Eastern Cooperative Oncology Group (ECOG) performance status (PS) of ≤2 if aged ≥18 years^34^ and Karnofsky or Lansky PS ≥60% if aged ≥16 years or 12 to <16 years^35^, respectively (see Supplementary Tables 5 and 6 for details of PS scoring systems). All patients were to receive either peripheral blood or bone marrow allo-HSCT for hematologic malignancy from unrelated donors who were 8 of 8 or 7 of 8 human leukocyte antigen (HLA)-matched (a single allele mismatch at HLA-A, HLA-B and HLA-C, and HLA-DRB1 was permitted). A total of 441 patients were screened for eligibility. After screening, 343 patients were randomly assigned 1:1 to receive vedolizumab (174 patients) or placebo (169 patients) treatment. Randomization was stratified by age (patients aged ≥18 years or aged 12 to <18 years); HLA match (8 of 8 versus 7 of 8); conditioning regimen intensity (myeloablative conditioning (MAC) versus reduced intensity conditioning (RIC)); and anti-thymocyte globulin (ATG) use (with versus without ATG). Patients received either vedolizumab 300 mg or placebo intravenously on day −1 and days +13, +41, +69, +97, +125 and +153 after allo-HSCT in addition to standard GVHD prophylaxis (CNI plus methotrexate or mycophenolate mofetil). Nine patients did not receive study treatment, five were randomized to vedolizumab and four were randomized to placebo treatment.

**Fig. 1:**
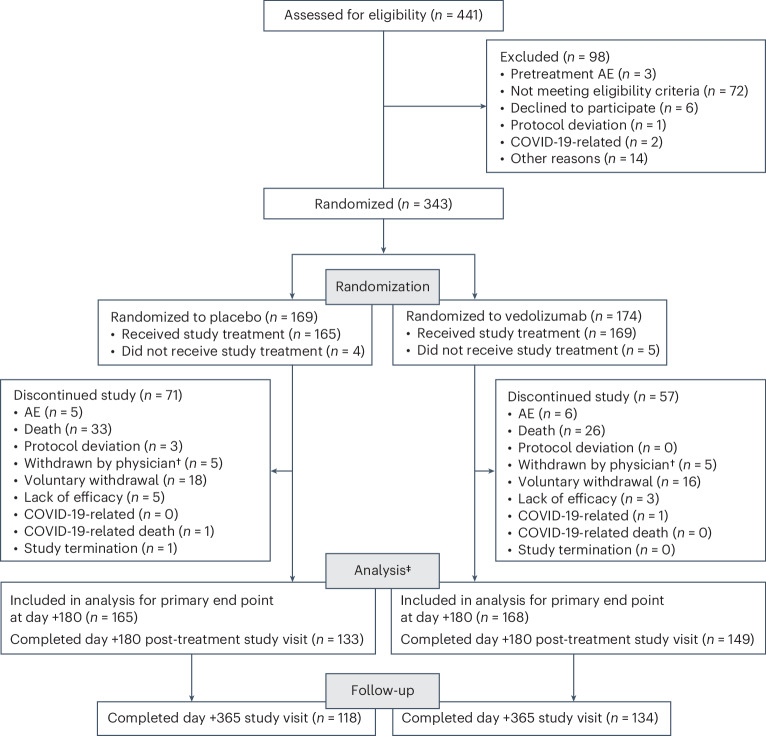
Eligibility, randomization and follow-up. Discontinuation of the study refers to all patients who discontinued before the end of the long-term follow-up safety survey period of the study, 6 months after the last dose of study treatment. ^†^Withdrawn by physician is noted as reason ‘other’. ^‡^Patients included in the analysis for efficacy end points per protocol were those who received ≥1 dose of study treatment and also received allo-HSCT. One patient was randomized to receive vedolizumab but did not receive allo-HSCT; per protocol, this patient was not included in the analysis of efficacy end points but was included in the analysis of safety end points.

Of 334 patients who received ≥1 dose of study treatment (analyzed for safety study end points), 333 also received allo-HSCT (analyzed for efficacy study end points), 168 in the vedolizumab group and 165 in the placebo group. For patients discontinuing the study, reasons for discontinuation included death (26 out of 57 patients in the vedolizumab group and 34 out of 71 in the placebo group), withdrawal by the patient (16 versus 18) and adverse events (AEs; 6 versus 5) (Fig. 1). Median (range) exposure to treatment was 40.0 (18.1–42.1) weeks for vedolizumab and 39.7 (18.1–42.3) weeks for placebo. In the vedolizumab group, patients received a mean (s.d.) of 5.4 (2.1) and median (range) 7.0 (1–7) treatment doses; 52.7% of patients in the vedolizumab group received all seven doses. A mean (s.d.) of 5.1 (2.3) and median (range) 7.0 (1–7) doses were received in the placebo group; 50.9% of patients in this group received all seven doses. Patient numbers were reduced to 60% of the planned sample size of 558 because of early enrollment termination owing to the impact of COVID-19 on recruitment. Consequently, more patients (*n* = 137, 41.1%) received ATG at baseline than the 25% planned.

Patient and transplant characteristics were balanced between treatment groups (Table 1 and Extended Data Table 1). The median age was 55.0 years (range, 16–74 years; 1 aged <18 years) and 62.8% were male. The most frequent underlying malignancies were acute myeloid leukemia (AML), myelodysplastic syndrome (MDS) and acute lymphoid leukemia (ALL). The conditioning regimen intensity was either MAC (52.4% in the vedolizumab group versus 53.9% in the placebo group) or RIC. GVHD prophylaxis (with or without ATG) was tacrolimus (TAC) + methotrexate (MTX; 42.3% versus 50.3%) or TAC + mycophenolate mofetil (MMF; 3.0% versus 3.0%); cyclosporine (CYS) + MTX (30.4% versus 23.0%) or CYS + MMF (14.3% versus 12.1%). The proportion of patients who received ATG prophylaxis was balanced between treatment groups: 42.3% (*n* = 71) in the vedolizumab group versus 40.0% (*n* = 66) in the placebo group; 57.7% versus 60.0% did not receive ATG.

**Table 1:** Baseline demographic, disease and transplant characteristics

Characteristic	Placebo (*N* = 165)	Vedolizumab (*N* = 168)
Median (min, max) age, years	55.0 (16–74)	53.0 (19–74)
Male, *n* (%)	106 (64.2)	103 (61.3)
Race, *n* (%)		
Asian	36 (21.8)	29 (17.3)
Black	2 (1.2)	3 (1.8)
White	114 (69.1)	121 (72.0)
Not reported	13 (7.9)	15 (8.9)
Primary disease, *n* (%)		
AML	72 (43.6)	73 (43.5)
ALL	24 (14.5)	29 (17.3)
Other myeloproliferative disorders	14 (8.5)	16 (9.5)
MDS	36 (21.8)	34 (20.2)
CML	5 (3.0)	6 (3.6)
CLL	0	1 (0.6)
Non-Hodgkin lymphoma	14 (8.5)	7 (4.2)
Hodgkin lymphoma	0	2 (1.2)
ECOG PS, *n* (%)		
0	65 (39.6)	64 (38.1)
1	86 (52.4)	89 (53.0)
2	13 (7.9)	14 (8.3)
3	0	1 (0.6)
4	0	0
Disease status at time of HSCT (AML and ALL), *n* (%)		
Complete remission 1	79 (84.0)	70 (71.4)
Complete remission >1	15 (16.0)	26 (26.5)
Other	0	2 (2.0)
Disease status at time of HSCT (CML), *n* (%)		
First chronic phase	0	3 (50.0)
Failing TKI	0	0
Accelerated phase or >1st chronic phase	2 (40.0)	1 (16.7)
Blast crisis	1 (20.0)	2 (33.3)
Progression	2 (40.0)	0
Cytogenetic risk (AML), *n* (%)		
Favorable	12 (18.5)	14 (20.6)
Intermediate	29 (44.6)	38 (55.9)
Unfavorable	24 (36.9)	16 (23.5)
Cytogenetic risk (MDS), *n* (%)		
Low	4 (11.8)	6 (18.2)
Intermediate 1	6 (17.6)	9 (27.3)
Intermediate 2	11 (32.4)	6 (18.2)
High	13 (38.2)	12 (36.4)
Stem cell source, *n* (%)		
Bone marrow	22 (13.4)	27 (16.1)
Peripheral blood	142 (86.6)	141 (83.9)
HLA compatibility,^a^ *n* (%)		
7 of 8 matched	19 (11.5)	22 (13.1)
8 of 8 matched	146 (88.5)	146 (86.9)
Conditioning regimen,^a^ *n* (%)		
MAC	89 (53.9)	88 (52.4)
RIC	76 (46.1)	80 (47.6)
ATG,^a^ *n* (%)		
With	66 (40.0)	71 (42.3)
Without	99 (60.0)	97 (57.7)
Background GVHD prophylaxis regimen, *n* (%)		
TAC + MTX	71 (43.0)	56 (33.3)
TAC + MTX + ATG	12 (7.3)	15 (8.9)
TAC + MMF	3 (1.8)	3 (1.8)
TAC + MMF + ATG	2 (1.2)	2 (1.2)
CYS + MTX	12 (7.3)	24 (14.3)
CYS + MTX + ATG	26 (15.8)	27 (16.1)
CYS + MMF	4 (2.4)	4 (2.4)
CYS + MMF + ATG	16 (9.7)	20 (11.9)
Other^b^	19 (11.5)	17 (10.1)

Data are from all randomized patients who received ≥1 dose of study treatment and received allo-HSCT. Baseline was defined as the last measurement on or before the day of the first dose of study treatment (day −1). CLL, chronic lymphocytic leukemia; CML, chronic myeloid leukemia; TKI, tyrosine kinase inhibitor.

^a^Information was collected at study randomization.

^b^Other TAC, CYS, MTX, MMF and ATG combinations.

### Engraftment

Neutrophil engraftment occurred in 165 patients in the vedolizumab treatment group and 160 patients in the placebo group. The median (range) time to neutrophil engraftment was 16.0 (8–35) days in the vedolizumab group and 15.0 (8–31) days in the placebo group. Platelet engraftment occurred in 159 patients in the vedolizumab group and 148 patients in the placebo group. The median (range) time to platelet engraftment was 18.0 (1–136) days in the vedolizumab group and 17.0 (0–233) days in the placebo group.

### Efficacy

#### Primary end point

**Fig. 2:**
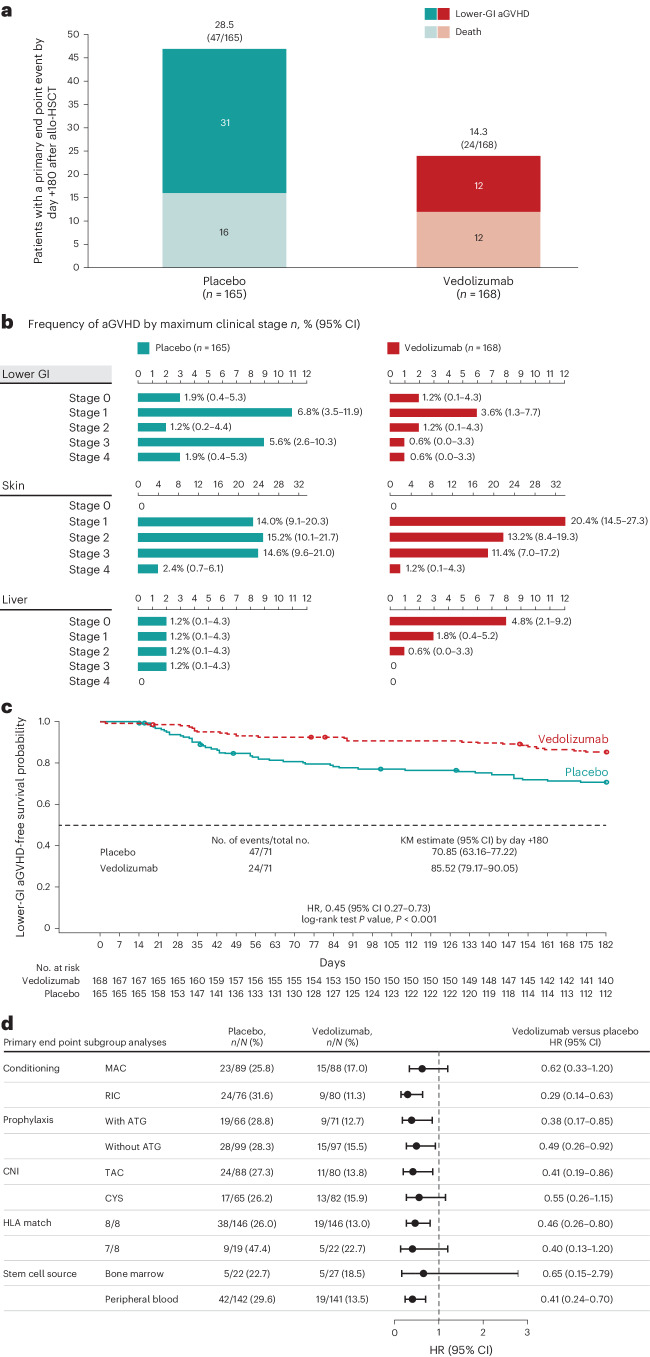
Lower-GI aGVHD-free survival by day +180 after allo-HSCT. Analysis included all randomized patients who received ≥1 dose of study treatment and received allo-HSCT. All statistical tests were two-sided. **a**, Graph shows number and proportion of patients with a lower-GI aGVHD event or death; censored for patients who had not had the lower-GI aGVHD event or died or had the event after a prespecified time, for example, last contact or day +180 after allo-HSCT, whichever occurred first. If a patient had a lower-GI aGVHD event and died due to any cause, including lower-GI aGVHD, the time to event was derived as the time to the first qualifying event (lower-GI aGVHD event). **b**, Frequency of lower-GI aGVHD by maximum clinical stages 0–4 by day +180 after allo-HSCT for patients in vedolizumab and placebo treatment groups and also the corresponding frequency of skin aGVHD and liver aGVHD in these treatment groups by maximum clinical stages 0–4 by day +180 after allo-HSCT. CI was based on the Clopper–Pearson method. **c**, KM estimate for the primary study end point lower-GI aGVHD-free survival from first study treatment (day −1) to lower-GI aGVHD event or death due to any cause. Red line shows the vedolizumab group; blue line shows the placebo group; open circles indicate censored patients. HR obtained via a Cox proportional hazards model with treatment group, stratified by randomization stratification factors: HLA match (7 of 8 or 8 of 8), conditioning regimen (MAC or RIC), ATG (with or without) and *P* value from a log-rank test (*P* = 0.0009). **d**, Forest plot of prespecified subgroup analyses for the primary study end point of lower-GI aGVHD-free survival by day +180 after allo-HSCT: conditioning regimen MAC or RIC, with or without ATG, CNI TAC or CYS, HLA match, and stem cell source peripheral blood or bone marrow. HRs plotted with 95% CIs were obtained via a Cox proportional hazards model with treatment group stratified by randomization strata. Results for the remaining prespecified subgroup analyses are shown in Extended Data Fig. 1.

**Extended Data Fig. 1:**
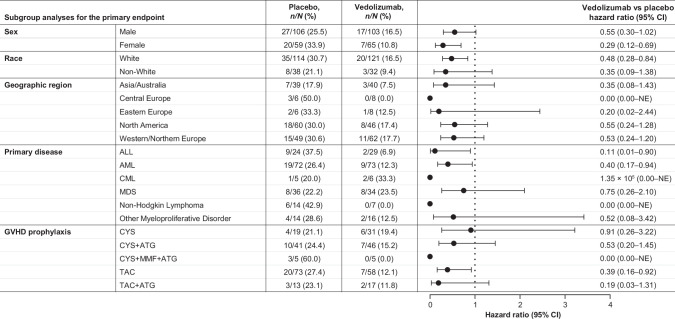
Subgroup analyses for the primary end point. Forest plot shows results of prespecified subgroup analyses for the primary efficacy end point lower-GI aGVHD-free survival by day+180 after allo-HSCT: sex; race; geographic region; primary disease; and GVHD prophylaxis, in addition to subgroup analyses reported in Fig. 2d. Analyses included all randomized patients who received ≥1 dose of study treatment and allo-HSCT; statistical tests were two-sided. Hazard ratios plotted with 95% confidence intervals were obtained via Cox proportional hazard model with treatment group stratified by randomization strata. ALL, acute lymphoid leukemia; AML, acute myeloid leukemia; ATG, anti-thymocyte globulin; CML, chronic myeloid leukemia; CYS, cyclosporin; MDS, myelodysplastic syndrome; MMF, mycophenolate mofetil; TAC, tacrolimus.

The primary study end point was lower-GI aGVHD-free survival by day +180 after allo-HSCT. There were 24 (14.3%) patients in the vedolizumab group with an event of lower-GI aGVHD or death by day +180 after allo-HSCT compared to 47 (28.5%) patients in the placebo group (Fig. 2a). The frequency of lower-GI aGVHD by maximum clinical stage (see Supplementary Table 1 for a description of clinical staging of aGVHD^9^) is shown in Fig. 2b for each treatment group, with four cases of stage 2–4 lower-GI aGVHD in the vedolizumab group compared to 14 cases observed in those who received placebo. The Kaplan–Meier (KM) estimate for lower-GI aGVHD-free survival by day +180 was 85.5% (95% confidence interval (CI) 79.2–90.1) for the vedolizumab group and 70.9% (63.2–77.2) for the placebo group (Fig. 2c). The risk of a lower-GI aGVHD event or death by day +180 after allo-HSCT was 55% less in the vedolizumab group compared to the placebo group (hazard ratio (HR) 0.45, 95% CI 0.27–0.73; *P* < 0.001). Results were consistent for sensitivity analyses of the primary end point (Table 2), including events occurring within a 7-day time frame at day +187 after allo-HSCT, stratified log-rank tests by randomization stratification factors, analysis with corrected stratification information, competing risk analysis and an analysis excluding aGVHD events graded stage 0 or unknown. By day +180 after allo-HSCT, 23 patients (13.7%) in the vedolizumab group versus 43 (26.1%) in the placebo group had an event of death or lower-GI aGVHD (when aGVHD events graded stage 0 or unknown were excluded) (HR 0.47, 95% CI 0.28–0.78; *P* = 0.0029). In subgroup analyses of the primary end point (Fig. 2d and Extended Data Fig. 1), HRs consistently favored vedolizumab over placebo regardless of HLA match, conditioning regimen intensity, use of ATG or stem-cell source (bone marrow or peripheral blood). The overall incidence of upper-GI aGVHD, skin aGVHD and aGVHD in the liver by day +180 after allo-HSCT was similar between treatment groups (Supplementary Table 7).

**Table 2:** Sensitivity analyses for the primary end point

Sensitivity analyses	Placebo (*N* = 165)		Vedolizumab (*N* = 168)		*P* value^b^	HR (95% CI)^c^
	Event, *n* (%)^a^	KM estimated survival, %	Event, *n* (%)^a^	KM estimated survival, %		
**Primary analysis**	47 (28.5)	70.9	24 (14.3)	85.5	0.0009	0.45 (0.27–0.73)
Sensitivity 1: using day +187 window^d^	48 (29.1)	70.2	24 (14.3)	85.5	0.0006	0.44 (0.27–0.71)
Sensitivity 2: by stratified log-rank test^e^	47 (28.5)	70.9	24 (14.3)	85.5	0.00096	0.45 (0.27–0.73)
Sensitivity 3: by competing risk analysis^f^	31 (18.8)	19.2 (cumulative incidence, %)	12 (7.1)	7.2 (cumulative incidence, %)	0.0012^f^	NA
Sensitivity 4: with corrected stratification information^g^	47 (28.5)	70.9	24 (14.3)	85.5	0.0009	0.45 (0.27–0.73)
Sensitivity 5: with clinical stage 0 and stage unknown removed^h^	43 (26.1)	73.3	23 (13.7)	86.1	0.0029	0.47 (0.28–0.78)

Prespecified sensitivity analyses for the primary end point of lower-GI aGVHD-free survival by day +180 after allo-HSCT, which included day +187 window, stratified log-rank test, competing risk analysis and corrected stratification information. Analyses included all randomized patients who received ≥1 dose of study treatment and received allo-HSCT. All statistical tests were two-sided.

^a^Number (%) of patients with an observed event/relapse/death (as applicable), whichever occurred first, from first study treatment dose (day −1) through day +180 or day +365. Full analysis set includes all randomized patients who received ≥1 dose of study treatment and received allo-HSCT.

^b^*P* value for the comparison between vedolizumab and placebo was obtained by log-rank test, unless otherwise stated.

^c^HR was obtained via a Cox proportional hazards model with treatment group, stratified by randomization strata: HLA match (7 of 8 or 8 of 8), conditioning regimen (MAC or RIC), ATG (with or without), unless otherwise stated.

^d^Sensitivity analysis 1, analysis of events occurring within a 7-day window at day +187 after allo-HSCT.

^e^Stratified log-rank test in sensitivity analysis 2 used to compare treatment groups stratified by randomization strata: HLA match (7 of 8 or 8 of 8), conditioning regimen (MAC or RIC), ATG (with or without).

^f^Competing risk is death in sensitivity analysis 3; *P* value for comparison of vedolizumab with placebo was obtained by Gray’s test.

^g^Sensitivity analysis 4 was performed using corrected stratification information for HLA match (7 of 8 or 8 of 8), conditioning regimen (MAC or RIC), ATG (with or without) collected after randomization.

^h^Sensitivity analysis 5 was an additional sensitivity analysis performed using an alternative primary end point definition excluding the investigator-indicated lower-GI aGVHD events with clinical stage as 0 or unknown in a post hoc fashion.

#### Secondary end points

**Fig. 3:**
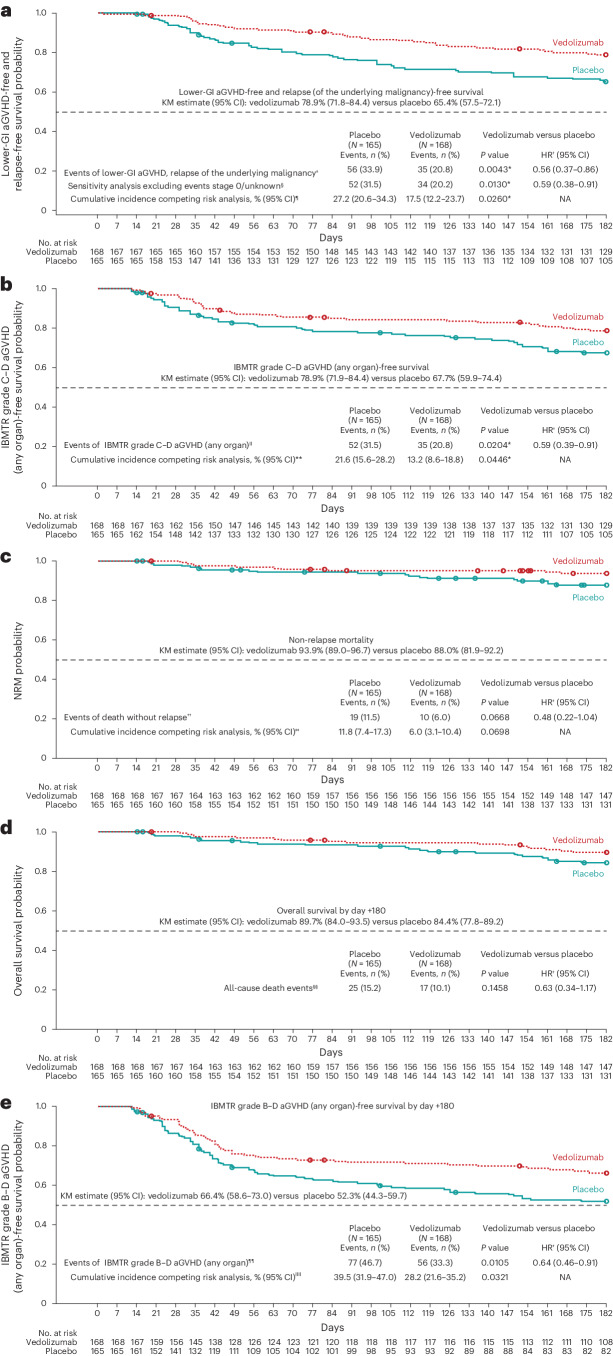
Key secondary efficacy end points by day +180 after allo-HSCT. **a**–**e**, KM estimates for the secondary efficacy end points. Analyses included all randomized patients who received ≥1 dose of study treatment and allo-HSCT. In the fixed-sequence hierarchical testing procedure, once 1 efficacy end point was not significant (*P* ≥ 0.05), testing of subsequent end points was not performed. *P* values were obtained using a log-rank test unless otherwise stated. All statistical tests were two-sided. *P value is significant for vedolizumab versus placebo. ^†^HR and 95% CI values were obtained from a Cox proportional hazards model with treatment group stratified by randomization strata: HLA match (7 of 8 or 8 of 8), conditioning regimen (MAC or RIC) and ATG (with or without). ^‡^Time to first documented lower-GI aGVHD, relapse of underlying malignancy or death from any cause. ^§^Sensitivity analysis, excluding lower-GI aGVHD events classified as clinical grade 0 or unknown. ^¶^NRM was a competing risk in this competing risk sensitivity analysis; *P* value for comparison of vedolizumab with placebo was obtained by a Gray’s test. ^‖^Time to first documented IBMTR grade C–D aGVHD (any organ) or death from any cause. ^**^Death and relapse were competing risks in this sensitivity analysis; an event was defined as IBMTR grade C–D aGVHD (any organ) or death. *P* value was obtained by a Gray’s test. ^††^Death from first dose of study treatment without occurrence of a relapse. ^‡‡^Relapse was a competing risk in this sensitivity analysis; NRM was the time from first study treatment to death without occurrence of a relapse; *P* value was obtained by a Gray’s test. ^§§^Overall survival by day +180 was the analysis of the time from the first dose of study treatment to death from any cause. All deaths were defined as events in this analysis. ^¶¶^Time to first documented IBMTR grade B–D aGVHD (any organ) or death from any cause. ^‖‖^Death and relapse were competing risks in this sensitivity analysis; an event was defined as IBMTR grade B–D aGVHD (any organ) or death. *P* value was obtained by a Gray’s test.

The KM estimates for the five key secondary end points analyzed at day +180 after allo-HSCT are shown in Fig. 3.

**Extended Data Fig. 2:**
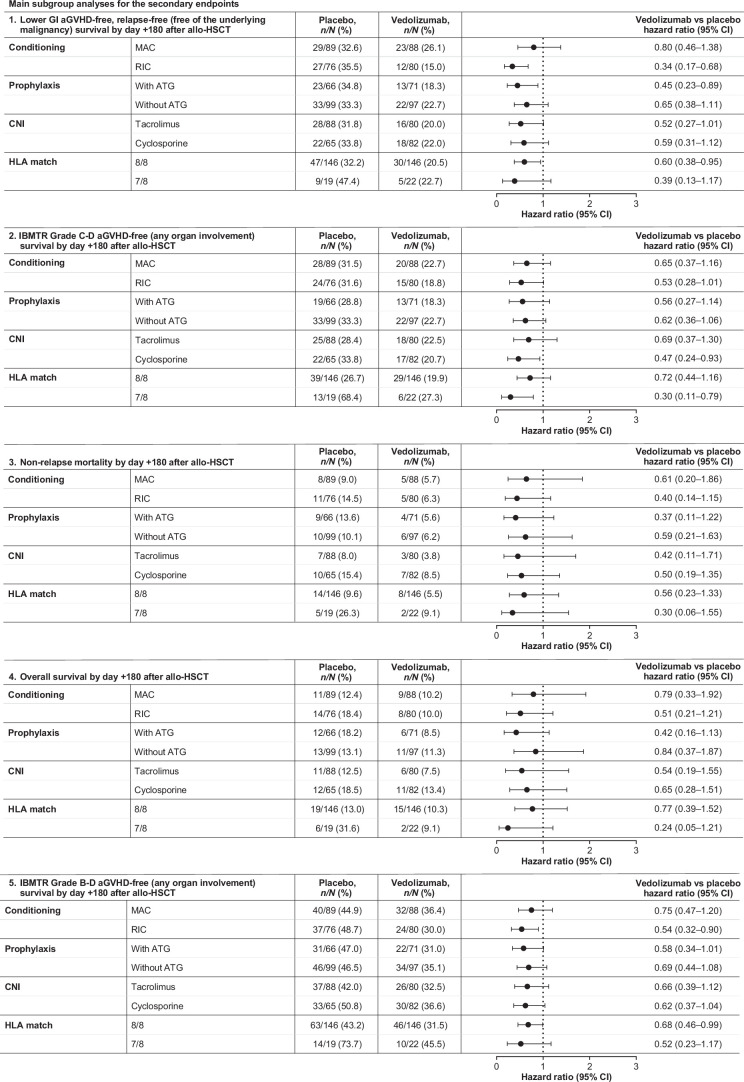
Subgroup analyses for the secondary end points. Forest plots show results of prespecified subgroup analyses for the secondary efficacy end points: conditioning regimen MAC or RIC; with or without ATG; CNI tacrolimus or cyclosporine; HLA match; and stem cell source peripheral blood or bone marrow. Analyses included all randomized patients who received ≥1 dose of study treatment and allo-HSCT; statistical tests were two-sided. Hazard ratios plotted with 95% confidence intervals were obtained via Cox proportional hazard model with treatment group stratified by randomization strata. Allo-HSCT, allogeneic hematopoietic stem cell transplantation; ATG, anti-thymocyte globulin; aGVHD, acute graft-versus-host disease; CNI, calcineurin inhibitor; HLA, human leukocyte antigen; MAC, myeloablative conditioning; RIC, reduced intensity conditioning.

There was a statistically significant difference favoring vedolizumab over placebo for lower-GI aGVHD-free and relapse of the underlying malignancy-free survival by day +180 after transplant. The KM estimated survival for this end point was 78.9% for the vedolizumab treatment group versus 65.4% for the placebo group. Events of lower-GI aGVHD, relapse or death for this end point occurred in 11, 18 and 6 patients, respectively from the vedolizumab group (total of 35, 20.8%) and 31, 13 and 12 (total of 56, 33.9%) in the placebo group (HR 0.56, 95% CI 0.37–0.86; *P* = 0.0043). A statistically significant treatment difference favoring vedolizumab for this end point was also maintained after a sensitivity analysis excluding stage 0 and unknown lower-GI aGVHD events (HR 0.59, 95% CI 0.38–0.91; *P* = 0.0130) (Fig. 3). The secondary end point of IBMTR grade C–D aGVHD of any organ-free survival by day +180 (see Supplementary Table 3 for description of aGVHD severity grading using the IBMTR severity index), also demonstrated a statistical difference between vedolizumab and placebo treatment groups. The KM estimated survival for this end point was 78.9% for vedolizumab the treatment group versus 67.7% in the placebo group. Events of grade C–D aGVHD of any organ or death counted for this end point occurred in 35 patients (20.8%) receiving vedolizumab versus 52 (31.5%) receiving placebo (HR 0.59, 95% CI 0.39–0.91; *P* = 0.0204). In a competing risk analysis (death and relapse as competing risks), cumulative incidence of IBMTR grade C–D aGVHD by day +180 was lower for the vedolizumab group (13.2%, 95% CI 8.6–18.8) than the placebo group (21.6%, 95% CI 15.6–28.2; *P* = 0.0446) (Fig. 3). Secondary end point sensitivity analyses (Supplementary Table 8) and subgroup analyses (Extended Data Fig. 2) showed consistent results with decreased risk in the vedolizumab group compared to the placebo treatment group. The secondary end point of non-relapse mortality (NRM) by day +180 did not meet statistical significance, with 10 patients (6.0%) in the vedolizumab group and 19 (11.5%) in the placebo group (HR 0.48, 95% CI 0.22–1.04; *P* = 0.0668) dying of non-relapse causes. Following the hierarchical statistical testing procedure, the subsequent fourth and fifth secondary end points were not tested for statistical significance. The KM estimate for the fourth secondary end point of overall survival was 89.7% for the vedolizumab treatment group and 84.4% in the placebo group. All-cause deaths by day +180 counted for this analysis occurred in 17 patients (10.1%) in the vedolizumab group and 25 (15.2%) in the placebo group (HR 0.63, 95% CI 0.34–1.17; *P* = 0.1458). For the fifth secondary end point of IBMTR grade B–D aGVHD of any organ-free survival by day +180, KM estimated survival was 66.4% for the vedolizumab treatment group and 52.3% in the placebo group. Grade B–D aGVHD events in any organ counted for this end point occurred in 47 patients (28.0%) in the vedolizumab group and 64 (38.8%) in the placebo group with deaths also counted in 9 and 13 patients in the vedolizumab and placebo groups, respectively (HR 0.64, 95% CI 0.46–0.91; *P* = 0.0105).

#### Exploratory efficacy end points

Results for the main exploratory end points at day +180 and day +365 after transplant are summarized (Extended Data Tables 3 and 4). The cumulative incidence of all chronic GVHD events by day +180 was 20.7% (95% CI 14.8–27.2) in the vedolizumab group versus 21.9% (95% CI 15.8–28.6) in the placebo group (death and relapse as competing risks; nominal *P* = 0.7555). Chronic GVHD requiring systemic immunosuppression by day +180 occurred in three (1.8%) patients in the vedolizumab group (severity was moderate in two patients and severe in one) and four (2.4%) in the placebo group (one mild, two moderate and one patient had severe chronic GVHD) (Extended Data Table 3). KM estimates for GVHD (any organ)-free and relapse (of the underlying malignancy)-free survival by day +180 were 80.1% in the vedolizumab group and 69.7% in the placebo group; events for this end point occurred in 33 (19.6%) of patients in the vedolizumab group and 49 (29.7%) in the placebo group (HR 0.61, 95% CI 0.39–0.96; nominal *P* = 0.0243). Events of clinical stage 2–4 lower-GI aGVHD or death by day +180 occurred in fewer patients in the vedolizumab group (19, 11.3%) than in the placebo group (33, 20.0%) (HR 0.52, 95% CI 0.29–0.91; nominal *P* = 0.0222). KM estimates for clinical stage 2–4 lower-GI aGVHD-free survival were 88.5% and 79.5%, respectively. By day +180 grade 2*–*4 aGVHD-free survival (per MAGIC criteria^10^, see Supplementary Table 4) also seemed to favor vedolizumab over placebo; KM estimates were 74.1% for vedolizumab and 63.3% for placebo, with events occurring in 43 (25.6%) and 59 (35.8%) patients, respectively (HR 0.67, 95% CI 0.45–0.99; nominal *P* = 0.0421). Frequency of lower-GI aGVHD by maximum MAGIC grade were also reported for each treatment group, with corresponding values for maximum MAGIC grade of skin and liver aGVHD (Extended Data Table 2).

Progression-free survival in vedolizumab and placebo treatment groups by day +180 were 83.1% (95% CI 76.5–88.0) versus 77.6% (95% CI 70.4–83.3), respectively. Cumulative incidence of all relapse and death events for time to relapse (of the underlying malignancy) by day +180 were similar across treatment groups 10.9% (95% CI 6.7–16.2) for vedolizumab versus 10.6% (95% CI 6.4–16.0) for placebo (death as a competing risk; nominal *P* = 0.9090). By day +180, there was no significant difference in relapse of the underlying malignancy between treatment groups, occurring in 18 (10.7%) patients from the vedolizumab group and 17 (10.3%) from the placebo group (HR 1.32, 95% CI 0.51–3.40; nominal *P* = 0.9821; Extended Data Table 3).

Consistent results were obtained for primary and secondary efficacy end points when these were assessed as exploratory study end points 1 year after allo-HSCT (Extended Data Table 4). By day +365 after allo-HSCT, 21.4% of patients in the vedolizumab group and 33.9% in the placebo group had an event of lower-GI aGVHD or death (HR 0.53, 95% CI 0.35–0.81; nominal *P* = 0.0041). KM estimates for lower-GI aGVHD-free survival 1 year after transplant were 78.1% for vedolizumab and 65.1% for placebo. Events of IBMTR grade C–D aGVHD of any organ or death by day +365 occurred in 47 (28.0%) of patients in the vedolizumab group and 59 (35.8%) of patients in the placebo group (HR 0.68, 95% CI 0.46–1.00; nominal *P* = 0.0709). Death without relapse occurred in 15 patients (8.9%) in the vedolizumab group and 25 (15.2%) in the placebo group (HR 0.49, 95% CI 0.25–0.95; nominal *P* = 0.0670). All-cause deaths by day +365 occurred in 28 patients (16.7%) in the vedolizumab group and 36 (21.8%) in the placebo group (HR 0.67, 95% CI 0.41–1.11; nominal *P* = 0.1741). IBMTR grade B–D aGVHD in any organ or death events occurred in 69 patients (41.1%) in the vedolizumab group and 82 (49.7%) in the placebo group (HR 0.71, 95% CI 0.52–0.99; nominal *P* = 0.0534). Incidence of relapse of the underlying malignancy at day +365 was also comparable between treatment groups occurring in 19.6% of patients in the vedolizumab group versus 13.3% for placebo (HR 2.13, 95% CI 0.97–4.65; nominal *P* = 0.2097; Extended Data Table 4).

### Safety

The safety analyses included 334 patients (169 patients in the vedolizumab group and 165 in the placebo group) who received ≥1 dose of study treatment and were assessed up to 18 weeks after the last dose of study treatment. Median (range) treatment exposure was 280.0 (127–295) days for the vedolizumab group (mean (s.d.) of 5.4 (2.05) doses) and 278.0 (127–296) days for the placebo group (mean 5.1 (2.25) doses). AEs of grade 3 or higher occurred in 92.3% of patients who received vedolizumab and 89.1% who received placebo (Table 3); the most frequent AEs of grade 3 or higher were anemia (29.6% versus 31.5%); neutropenia (31.4% versus 29.7%); febrile neutropenia (43.8% versus 42.4%); stomatitis (27.2% versus 26.7%); and decreased platelet count (21.9% versus 24.8%). Serious AEs occurred in 120 patients (71.0%) who received vedolizumab and 114 (69.1%) who received placebo (Extended Data Table 5). AEs led to treatment discontinuation in 44 (26.0%) versus 51 patients (30.9%) (Extended Data Table 6).

**Table 3:** Adverse events

Preferred term	Patients, *n* (%)	
	Placebo (*N* = 165)	Vedolizumab (*N* = 169)
Any AE, *n* (%)	165 (100)	169 (100)
Ten most common non-serious AEs (≥5% patients), *n* (%)		
Diarrhea	102 (61.8)	95 (56.2)
Stomatitis	90 (54.5)	90 (53.3)
Nausea	83 (50.3)	85 (50.3)
Pyrexia	62 (37.6)	80 (47.3)
Anemia	72 (43.6)	68 (40.2)
aGVHD in skin	65 (39.4)	67 (39.6)
Febrile neutropenia	54 (32.7)	67 (39.6)
Headache	58 (35.2)	58 (34.3)
Hypomagnesemia	52 (31.5)	60 (35.5)
Hypertension	54 (32.7)	54 (32.0)
Drug-related AE, *n* (%)	41 (24.8)	48 (28.4)
Grade ≥3 AE, *n* (%)	147 (89.1)	156 (92.3)
Drug-related grade ≥3 AE, *n* (%)	19 (11.5)	18 (10.7)
AE leading to study drug discontinuation, *n* (%)	51 (30.9)	44 (26.0)
Any serious AE, *n* (%)	114 (69.1)	120 (71.0)
Drug-related serious AE, *n* (%)	14 (8.5)	11 (6.5)
Serious AE leading to study drug discontinuation, *n* (%)	38 (23.0)	39 (23.1)
AEs leading to death, *n* (%)	27 (16.4)	21 (12.4)
AESIs		
Serious infection^a^	111 (67.3)	125 (74.0)
Three most frequent serious infections		
CMV reactivation	30 (18.2)	40 (23.7)
Pneumonia	14 (8.5)	13 (7.7)
Sepsis	12 (7.3)	9 (5.3)
PML^b^	2 (1.2)	3 (1.8)
Secondary malignancy^c^	16 (9.7)	7 (4.1)
Liver injury	69 (41.8)	68 (40.2)
Hypersensitivity/injection site reaction^d^	136 (82.4)	134 (79.3)
Leukopenia/lymphopenia	7 (4.2)	14 (8.3)
CMV infection	3 (1.8)	3 (1.8)
Grade 1	0	0
Grade 2	1 (0.6)	2 (1.2)
Grade 3	1 (0.6)	1 (0.6)
Grade 4	1 (0.6)	0
Grade 5	0	0
Reactivation of CMV	30 (18.2)	40 (23.7)
Grade 1	6 (3.6)	9 (5.3)
Grade 2	18 (10.9)	25 (14.8)
Grade 3	6 (3.6)	6 (3.6)
Grade 4	0	0
Grade 5	0	0
CMV viremia	3 (1.8)	1 (0.6)
Grade 1	0	0
Grade 2	2 (1.2)	1 (0.6)
Grade 3	1 (0.6)	0
Grade 4	0	0
Grade 5	0	0
CMV colitis	1 (0.6)	1 (0.6)
Grade 1	0	0
Grade 2	1 (0.6)	0
Grade 3	0	1 (0.6)
Grade 4	0	0
Grade 5	0	0
Post-transplant lymphoproliferative disorder	3 (1.8)	0
*Clostridioides* infections^e^	6 (3.6)	14 (8.3)

Data were from the safety analysis set, which included all patients who received ≥1 dose of study treatment. AE was defined as any AE newly occurring or worsening from the first dose up to 18 weeks after the last dose of study treatment. Serious AEs were defined as a serious AE newly occurring or worsening from the first dose of study treatment up to 18 weeks after the last dose of study treatment. AESIs prespecified in the study protocol were serious infections, PML, secondary malignancies, liver injury, hypersensitivity reactions, leukopenia/lymphopenia, CMV colitis and CMV reactivation.

^a^Defined as any infection or infestation event not classified as a preferred term of CMV colitis (CMV colitis experienced in one patient per group; 0.6%).

^b^Suspected PML cases were five patients with an AE of human polyomavirus infection; none of these were diagnosed as PML. One patient with AML relapse and subsequent additional therapy developed PML with a fatal outcome at ~6 months after the last dose of vedolizumab. An independent adjudication committee deemed the most probable cause of this event to be the immunosuppressive treatment for AML.

^c^Secondary malignancies excluding relapse of the primary disease.

^d^Including infusion-related reactions and injection site reactions.

^e^Patients with *C.* *difficile* infection, *C.* *difficile* colitis or *Clostridioides* colitis. No patient had more than one event of *C.* *difficile* infection, *C.* *difficile* colitis or *Clostridioides* colitis.

Table 3 lists serious infections among other AEs (serious and non-serious) prespecified as being of special interest (AESIs) in the study. Occurrence of post-transplant lymphoproliferative disease and *Clostridioides* infections are also reported in Table 3. AESIs included cytomegalovirus (CMV) colitis, which was reported in one patient from each treatment group (0.6% of patients in vedolizumab group 0.6% in the placebo group). Overall, CMV reactivation was reported in 23.7% of patients in the vedolizumab group and 18.2% in the placebo group. Most of the CMV reactivation events were grade 1 to grade 2 and none was above grade 3. The proportions of patients with grade 3 CMV reactivation were similar in both treatment groups. CMV infections were analyzed in subgroups of patients who received ATG prophylaxis or not (Supplementary Table 9). For those receiving ATG, grade ≥3 CMV infections occurred in seven patients (4.1%) in the vedolizumab group and six patients (3.6%) in the placebo group and serious CMV infections in seven (4.1%) versus three patients (1.8%), respectively. For patients treated without ATG, the frequency of grade ≥3 CMV infections was numerically lower in vedolizumab-treated versus placebo-treated patients (1 (0.6%) versus 3 (1.8%), respectively), one patient in the vedolizumab treatment group had a serious CMV infection. Other serious infections (excluding CMV colitis) occurred in 125 (74.0%) of patients receiving vedolizumab versus 111 (67.3%) receiving placebo. These are listed by infection type (Extended Data Table 7). The most common serious infections were CMV reactivation (23.7% versus 18.2%); pneumonia (7.7% versus 8.5%); sepsis (5.3% versus 7.3%); and bacteremia (4.7% versus 5.5%) (Table 3). Serious abdominal and GI infections occurred in eight patients receiving vedolizumab (4.7%) and three receiving placebo (1.8%). *Clostridioides* infections occurred in 14 (8.3%) patients in the vedolizumab treatment group and six (3.6%) patients in placebo treatment group; of these 2.4% of patients in each treatment group had *Clostridioides* colitis (*C.* *difficile* colitis or *Clostridioides* colitis). For safety end points, statistical analyses were not adequately powered for comparisons between treatment groups. There were five patients with an AE of human polyomavirus infection; none of these was diagnosed as progressive multifocal leukoencephalopathy (PML). One patient with AML relapse and subsequent additional therapy developed PML, with a fatal outcome ~6 months after the last dose of vedolizumab. An independent adjudication committee deemed the most probable cause of this event to be the immunosuppressive treatment for AML. Secondary malignancies occurred in seven patients (4.1%) in the vedolizumab group and 16 (9.7%) in the placebo group. Post-transplant lymphoproliferative disease occurred in three patients (1.8%) in the placebo group only (Table 3).

Overall, 48 patients died during the period from first dose of study treatment to 18 weeks after last dose: 21 (12.4%) in the vedolizumab group and 27 (16.4%) in the placebo group. Leading causes of death were multiple organ dysfunction syndrome (3.0% versus 1.8%); AML recurrence (0.6% versus 2.4%); respiratory failure (1.8% versus 1.2%); pneumonia (1.2% versus 1.2%); and sepsis (0.0% versus 1.8%). Intestinal aGVHD was listed as cause of death in 0.0% versus 1.2% patients, aGVHD in liver (0.6% versus 0.6%) and aGVHD (0.6% versus 0.0%). An additional 17 patients died during the period from 18 weeks post-treatment to 12 months after HSCT: eight in the vedolizumab group and nine in the placebo group (Extended Data Table 8).

## Discussion

This international, phase 3, randomized double-blinded trial demonstrated significant improvement in lower-GI aGVHD-free survival by day +180 after allo-HSCT with vedolizumab, compared to placebo, when added to standard CNI-based GVHD prophylaxis in patients receiving unrelated donor allo-HSCT. Fewer patients receiving vedolizumab treatment had lower-GI aGVHD or death by day +180 compared to placebo and there were no events of GI GVHD-related deaths in the group receiving vedolizumab. Vedolizumab was consistently favored over placebo regardless of HLA match, conditioning regimen intensity, CNI (TAC or CYS), use of ATG or stem-cell source (peripheral blood or bone marrow). The study met its primary end point despite reduced enrollment rates due to the COVID-19 pandemic (60% of planned sample size, 71 of 148 planned events and 41% versus 25% planned patients receiving ATG); the difference in outcomes was driven by a significant reduction in lower-GI aGVHD. The primary end point was focused on lower-GI aGVHD due to its impact on morbidity and mortality as well as recent evidence from a Center for International Blood and Marrow Transplant Research registry analysis of over 8,000 allo-HSCT recipients showing that upper-GI aGVHD, in isolation or combination, did not have a significant prognostic impact on overall outcomes^36^. The incidence of isolated upper-GI aGVHD recorded without concurrent lower-GI aGVHD was low and there was no difference between treatment groups. Treatment-related differences in the overall incidence of lower-GI aGVHD observed were driven by the reduced frequency of lower-GI aGVHD in the vedolizumab group relative to the placebo group.

Secondary efficacy end points of lower-GI aGVHD-free and relapse of the underlying malignancy-free survival by day +180 and IBMTR grade C–D aGVHD-free survival by day +180 also significantly favored vedolizumab over placebo. Differences between treatment groups did not reach statistical significance for the three subsequent secondary efficacy end points of NRM by day +180 (10 patients (6.0%) in the vedolizumab group and 19 (11.5%) in the placebo group), overall survival by day +180 (89.7% versus 84.4%, respectively) and IBMTR grade B–D aGVHD of any organ-free survival by day +180 (66.4% versus 52.3%, respectively). Lower-GI aGVHD-free survival continued to be significantly higher in the vedolizumab group versus placebo at 1 year after allo-HSCT. Fifteen (8.9%) patients in the vedolizumab group and 25 patients (15.2%) in the placebo group died without relapse 1 year after transplant (HR 0.49, 95% CI 0.25–0.95; nominal *P* = 0.0670).

No new safety signals were identified for intravenous vedolizumab 300 mg when added to standard CNI-based GVHD prophylaxis in patients undergoing allo-HSCT. There were no clinically relevant differences in safety outcomes between the vedolizumab and placebo treatment groups and the AEs observed were consistent with the population under study. Serious AEs occurred in a similar proportion of patients in each treatment group; 71% for vedolizumab and 69% for placebo and the proportion of serious AEs designated as treatment-related was also comparable across treatment groups (7% in the vedolizumab group and 9% in the placebo group). The incidence of serious abdominal and GI infections in vedolizumab-treated patients was 5% compared to 2% receiving placebo. The proportion of patients with *Clostridioides* colitis (including *C.* *difficile* colitis) was also similar between treatment groups (2.4% in the vedolizumab group and 2.4% in placebo group); however, further studies would be necessary to fully evaluate the impact of targeting α_4_β_7_-integrin lymphocyte homing on the risk of *C.* *difficile* infection in the setting of GVHD prophylaxis. CMV colitis (serious or non-serious) occurred in one patient (0.6%) in each treatment group. There was no significant difference in relapse incidence between treatment groups.

The dose of vedolizumab chosen for this study was based on safety, efficacy, engraftment and pharmacokinetics (PK) data from a previous phase 1b, open-label study in patients undergoing unrelated donor allo-HSCT^33^ as well as data from patients with inflammatory bowel disease. Although there are no data on PK presented here to allow an evaluation of exposure–response in vedolizumab-treated patients, a study to characterize the population PK of vedolizumab used for prevention of aGVHD in patients undergoing unrelated donor allo-HSCT has been conducted using pooled clinical data (T. Waterhouse et al., manuscript in preparation). This analysis showed that vedolizumab PK characteristics and identified covariates were similar to those of the previous population PK model in patients with inflammatory bowel disease^37^.

While vedolizumab seemed to prevent lower-GI aGVHD in this study, relevance of the findings in this trial are in question after the results of the CTN 1703 study, which showed the superiority of post-transplant cyclophosphamide/TAC/MMF (PtCy) as GVHD prevention when compared to standard TAC/MTX^38^ in patients undergoing RIC transplant from well-matched peripheral blood stem cell grafts. In contrast, our study population also included patients receiving MAC, ATG products and bone marrow grafts. We note that the lack of information captured on ATG treatment used, including ATG product, dose and regimen, is a limitation of our study. Standard GVHD prevention after MAC is controversial given the added toxicity of PtCy to MAC and results of past trials showing comparable outcomes^39^. The subgroup of patients receiving ATG products is especially notable given the benefit of vedolizumab observed and the prevalent use of ATG products, especially in Europe where ATG products in combination with CNI-based regimens remains the standard of care based on efficacy in the prevention of chronic GVHD^40,41^, validated by long-term follow-up results^42^. In addition, a recently completed phase 2b randomized trial comparing PtCy/CYS to ATG/CYS in RIC well-matched peripheral blood HSCT showed comparable outcomes^43^. Therefore, the addition of vedolizumab to a standard CNI-based platform remains relevant. Adding vedolizumab to PtCy is the subject of ongoing research^44^, although such trials focusing on aGVHD prevention will be increasingly difficult to conduct given the diminishing number of events observed as care is improved.

Although this was a large randomized prospective trial, there are some limitations to mention in addition to those already discussed in terms of ATG use and the emergence of post-transplant cyclophosphamide. The diagnosis of aGVHD in this trial relied solely on the clinical judgment of the investigator rather than adjudication by a blinded central committee. While background GVHD prophylaxis was dictated by the trial, there was certainly significant heterogeneity of patients and transplant practices across the participating centers, including specific conditioning regimens, infectious disease prevention and supportive care. As discussed, enrollment was terminated prematurely due to the effect of the COVID-19 pandemic, possibly precluding more definitive interpretation of other end points. In addition, follow-up was only through 12 months after HSCT, thereby limiting comprehensive conclusions that could be drawn regarding any impact on the incidence of chronic GVHD and other long-term outcomes.

The reduction of lower-GI aGVHD observed in this study likely results from disrupted α_4_β_7_ integrin-mediated homing and transmigration of alloreactive T cells to the intestinal mucosa in vedolizumab-treated patients^30,33^. Notably, there was no additional increase in AEs or increase in relapse rates. Given the morbidity and mortality attributed to lower-GI aGVHD^15^ and lack of specific gut-targeted prophylactic therapies, the benefit of the routine addition of vedolizumab to standard HSCT platforms is potentially practice changing.

## Methods

### Study design

The study was a double-blind, placebo-controlled, randomized, multicenter, phase 3 trial (NCT03657160, EudraCT number 2018-002141-11) evaluating the efficacy and safety of intravenous vedolizumab added to standard GVHD prophylaxis with a CNI and either MTX or MMF in patients undergoing allo-HSCT (enrollment was expanded to include non-adult patients (aged ≥12 years) at selected sites). Enrollment ran from 6 February 2019 through 9 May 2022 at 95 centers: 19 in North America, 7 in South America, 45 in Europe and 24 in Asia/Australia (for study locations, see Supplementary Table 10). The study consisted of a 30-day pre-transplantation screening period, a 155-day treatment period, an end-of-treatment visit at day +180 after allo-HSCT (or an early-termination visit for patients who discontinued treatment) and a post-treatment follow-up period for assessment of safety, development of GVHD and overall survival completed by 1 year after allo-HSCT or until death/study withdrawal or study termination. After study enrollment and screening, eligible patients were randomized in a 1:1 ratio to receive vedolizumab or placebo. Treatment assignment was carried out using an interactive response technology system within 2 days before the first dose of study treatment. All study site personnel and patients were blinded to treatment assignments during the study, except those directly involved with study drug preparation. Randomization was stratified by age (patients aged ≥18 years or aged 12 to <18 years); HLA match (8 of 8 versus 7 of 8); conditioning regimen intensity (MAC versus RIC); and ATG treatment status (with versus without ATG).

### Ethics statement

The study was performed in accordance with the Declaration of Helsinki and the International Conference on Harmonisation Good Clinical Practice guidelines. Local institutional review boards of all participating centers approved the protocol and all patients, or their legal guardians, provided written informed consent. An independent data monitoring committee reviewed interim efficacy and safety results.

### Patients

Study inclusion and exclusion criteria were assessed by the study investigators during patient screening. Demographic information was obtained during screening and included information on age, sex, race and ethnicity. Information on biological sex was assigned by the study investigator or designee according to medical notes. The main inclusion criteria for study participants were age of at least 12 years, sex male or female, weighing ≥30 kg at randomization and candidates for allo-HSCT sourced from either unrelated peripheral blood or bone marrow as treatment for hematologic malignancies or myeloproliferative disorders. After DNA-based matching, eligible patients were 8 of 8 or 7 of 8 HLA-matched (a single allele or antigen mismatch at HLA-A, HLA-B and HLA-C, and HLA-DRB1 was permitted) and also met local institutional eligibility criteria for allo-HSCT. Those included in the study (Fig. 1) also had either an ECOG PS ≤2 (if aged ≥18 years) or a Karnofsky PS ≥60% (if aged ≥16 years) or a Lansky PS ≥60% (if aged 12 to <16 years) (Supplementary Tables 5 and 6)^35^.

The main study exclusion criteria included previous allo-HSCT; patients planning to undergo umbilical cord blood transplant, allo-HSCT for non-malignant hematological disorders or patients planned to receive ex vivo T cell-depleted hematopoietic stem cells or post-transplant cyclophosphamide were also excluded. Previous treatment with T cell-depleting antibodies except ATG (ATG-Fresenius (ATG-F) or thymoglobulin) within 4 months of the first dose of study treatment was also prohibited. Patients previously diagnosed or treated for another malignancy within 2 years of the first dose of study treatment or with evidence of residual disease were ineligible for the study, as were patients with any active cerebral/meningeal disease (including central nervous system involvement of the primary disease) or symptoms/history of PML. Patients with an active systemic infection, active CMV colitis, active *C.* *difficile* infection or other intestinal pathogen were also ineligible for the study.

### Treatment

Patients were to receive 7 intravenous doses of study treatment over a 155-day period starting on day −1 before allo-HSCT and then on days +13, +41, +69, +97, +125 and +153 after allo-HSCT, with a primary end point assessment on day +180 after allo-HSCT or early-termination visit. Post-treatment follow-up to assess safety, relapse, GVHD and overall survival continued up to 12 months after allo-HSCT. All patients were to receive standard GVHD prophylaxis comprising a CNI (CYS or TAC and MTX or MMF). ATG prophylaxis was permitted but was capped at ~25% of total study participants per protocol. At the investigator’s discretion, MMF could be used in place of MTX. Any other agent used for GVHD prophylaxis, apart from corticosteroids and ATG, was prohibited. Other prohibited concomitant medications included live vaccines (within 30 days of randomization ≥6 months after last dose of study treatment), immune-checkpoint inhibitors and any other biologic treatment, other than localized injections.

### End points

All randomized patients receiving ≥1 dose of study treatment and who underwent allo-HSCT were analyzed for efficacy end points. The primary end point was lower-GI aGVHD-free survival by day +180 after allo-HSCT, defined as the time from the first dose of study treatment (day −1) to the first clinically diagnosed presentation of lower-GI aGVHD or death from any cause, whichever occurred first. Only cases of lower-GI aGVHD or death by day +180 were counted for this analysis.

Key secondary end points evaluated at day +180 after allo-HSCT were lower-GI aGVHD-free and relapse-free survival; IBMTR severity index^11^ grade C–D aGVHD (any organ)-free survival; NRM; overall survival; and IBMTR grade B–D aGVHD (any organ)-free survival. The main exploratory efficacy end points included the primary and key secondary end points (assessed at 1 year after allo-HSCT). In addition, aGVHD outcomes were also assessed by MAGIC criteria^10^ (grade 2–4 aGVHD per MAGIC criteria-free survival and grade 3–4 aGVHD per MAGIC criteria-free survival) and frequency of aGVHD by maximum MAGIC criteria and organ involvement. Other exploratory end points assessed by day +180 and 1 year after allo-HSCT included: clinical stage 2–4 (ref. ^9^) lower-GI aGVHD-free survival; GVHD (any organ)-free and relapse (of the underlying malignancy)-free survival (where an event was defined as death or aGVHD grade 3–4 by modified Glucksberg criteria^9^ (Supplementary Table 2) or chronic GVHD requiring system immunosuppression or relapse); relapse of the underlying malignancy-free survival (progression-free survival); chronic GVHD-free survival; chronic GVHD requiring systemic immunosuppression-free survival; and incidence and duration of systemic immunosuppression for GVHD treatment. Incidence of relapse of the underlying malignancy by day +180 and 1 year after allo-HSCT was also assessed.

Safety and tolerability were assessed from the first dose to 18 weeks from the last study treatment by monitoring of vital signs, AEs, physical examinations and laboratory tests (chemistry, hematology and liver function). All patients who received ≥1 dose of the study treatment were analyzed for safety end points. No statistical inferences were made for safety analyses. AEs and serious AEs were coded using the Medical Dictionary for Regulatory Activities (v.24.0).

Engraftment was assessed as part of hematologic laboratory evaluations. Neutrophil engraftment was defined as neutrophil count >500 mm^−^^3^ for three consecutive days or >2,000 mm^−^^3^ for 1 day. Platelet engraftment was defined as a platelet count >20,000 cells per mm^3^ without transfusion.

### Statistical analysis

A sample size of approximately 558 patients (assuming an event rate of 34.1% for the placebo group and 21.8% for the vedolizumab group and 10% loss to follow-up for both groups) was planned to generate 148 primary efficacy events to detect a target HR of 0.59 between vedolizumab and placebo with 90% power for the primary end point based on a two-sided log-rank test at a significance level of 0.05.

#### Key trial amendments

Study enrollment was terminated early and the study completed on 9 May 2022, primarily due to low patient recruitment and enrollment during the COVID-19 pandemic (from February 2020). As a consequence, the end-of-treatment analysis was not performed as planned and the statistical analysis plan was amended to reflect this change. The end-of-study analysis (final analysis) was performed after the final database lock at the end of study. There were 19 patients with ≥1 significant COVID-19-related protocol deviation; of these, 9 (5.2%) were in the vedolizumab treatment group and 10 (5.9%) were in the placebo group. The sample size remained as planned after the prespecified interim adaptive sample size re-estimation using the promising zone design. One interim analysis for futility and sample size re-estimation was planned for this study. To control the overall type I error rate in the presence of planned interim analysis with sample size re-estimation, the 50% conditional power principle was implemented, which allowed sample size increase only when the unblinded interim results were promising or the conditional power was greater than 50% (refs. ^45–47^). Thus, the conventional unweighted test statistics and critical values were used in the final analysis without inflation of the type I error rate. Following the intent-to-treat principle, all randomized patients receiving ≥1 dose of study treatment and who underwent allo-HSCT were analyzed for efficacy end points. All patients who received ≥1 dose of the study treatment were analyzed for safety end points. All time-to-event end points were analyzed using log-rank tests. Cox proportional hazards models, stratified by randomization stratification factors, were fitted and HRs were reported. All dichotomous efficacy end points were analyzed using Cochran–Mantel–Haenszel tests for risk differences, stratified by randomization stratification factors. To control the overall type I error rate for the comparison between vedolizumab and placebo groups for the primary and key secondary efficacy end points in the final analysis, a fixed-sequence hierarchical testing procedure was used.

Prespecified end point sensitivity analyses included events occurring within a 7-day window at day +187 after allo-HSCT, stratified log-rank tests by randomization stratification factors, analysis with corrected stratification information and competing risk analysis (except overall survival end points). Additional post hoc sensitivity analyses included an alternative primary end point definition, excluding investigator-indicated lower-GI aGVHD events with clinical stage as 0 or unknown. Subgroup analyses were applied according to prespecified subpopulations of interest and included: conditioning regimen intensity; prophylaxis with or without ATG; CNI (TAC or CYS); HLA match; stem cell source (peripheral blood or bone marrow); sex (male or female); race; geographic region; primary disease; and GVHD prophylaxis.

### Reporting summary

Further information on research design is available in the Nature Portfolio Reporting Summary linked to this article.

## Online content

Any methods, additional references, Nature Portfolio reporting summaries, source data, extended data, supplementary information, acknowledgements, peer review information; details of author contributions and competing interests; and statements of data and code availability are available at 10.1038/s41591-024-03016-4.

### Supplementary information

Supplementary Information Supplementary Tables 1–10.

Reporting Summary

## Figures and Tables

**Extended Data Table 1:**
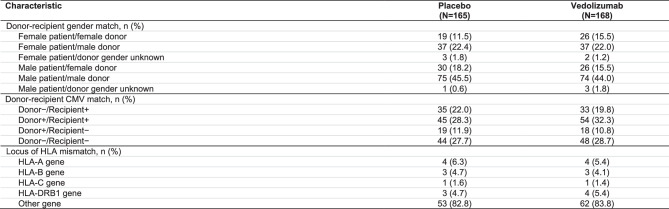
Baseline donor characteristics

Analysis included all randomized patients who received ≥1 dose of study treatment and allo-HSCT.

Baseline was defined as the last measurement on or before the day of the first dose of study treatment (day −1).

allo-HSCT, allogeneic hematopoietic stem cell transplantation; CMV, cytomegalovirus; HLA, human leukocyte antigen.

**Extended Data Table 2:**
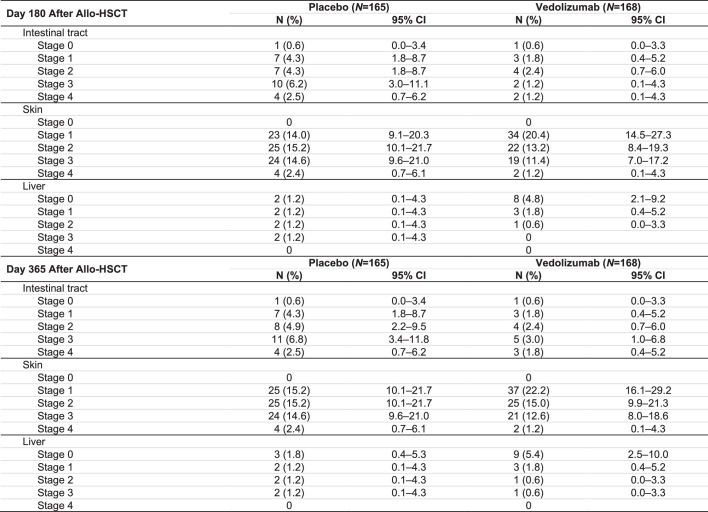
Frequency of aGVHD by organ involvement and maximum MAGIC criteria

Analysis included all randomized patients who received ≥1 dose of study treatment and allo-HSCT. Confidence interval (CI) was based on the Clopper-Pearson method.

Allo-HSCT, allogeneic hematopoietic stem cell transplantation; MAGIC, Mount Sinai Acute GVHD International Consortium (Harris AC, et al. Biol Blood Marrow Transplant 2016;22:4-10).

**Extended Data Table 3:**
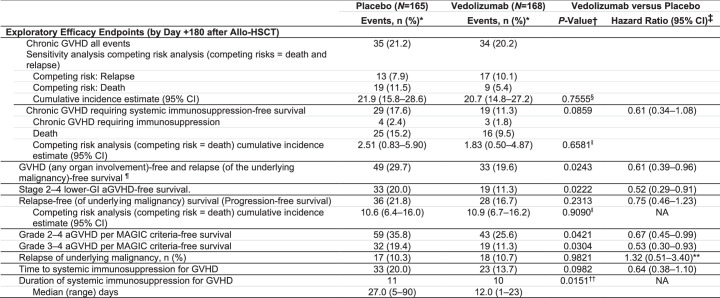
Results summary for main exploratory efficacy end points – by day +180

Results of the main exploratory study end points analyzed by day+180 after allo-HSCT. Analyses included all randomized patients who received ≥1 dose of study treatment and allo-HSCT. All statistical tests were two-sided.

* Number (%) of patients with an observed event/relapse/death (as applicable), whichever occurred first, from first study treatment dose (day −1) through day +180.

† Obtained via log-rank test unless otherwise stated.

‡ Obtained via Cox proportional hazards model with treatment group, stratified by randomization stratification factors: HLA match (7/8, 8/8), conditioning regimen (Myeloablative, Reduced Intensity), ATG (With, Without) unless otherwise stated.

§ Competing risk analysis with relapse and death as competing risks. *P*-value obtained by Gray’s test.

¶ GVHD and relapse-free survival was the time from first study treatment dose (day-1) to GVHD event/death/relapse, where an event is defined as death or aGVHD Grade 3–4 by modified Glucksberg criteria^9^ (Supplementary Table 2) or chronic GVHD requiring system immunosuppression or relapse.

‖ Competing risk analysis with death as competing risk. *P*-value obtained by Gray’s test.

**Hazard ratio obtained via Cox proportional hazards model with treatment group adjusting for complete remission status and randomization stratification factors: HLA match or mismatch (8/8 or 7/8), Conditioning Regimen (Myeloablative, Reduced Intensity), and ATG (With, Without).

††*P*-value for comparison between vedolizumab and placebo was obtained using Wilcoxon rank-sum test.

Allo-HSCT, allogenic hematopoietic stem cell transplantation; ATG, anti-thymocyte globulin; HLA, human leukocyte antigen; GVHD, graft-versus-host disease; MAGIC, Mount Sinai Acute GVHD International Consortium (Harris AC, et al. Biol Blood Marrow Transplant 2016;22:4-10).

**Extended Data Table 4:**
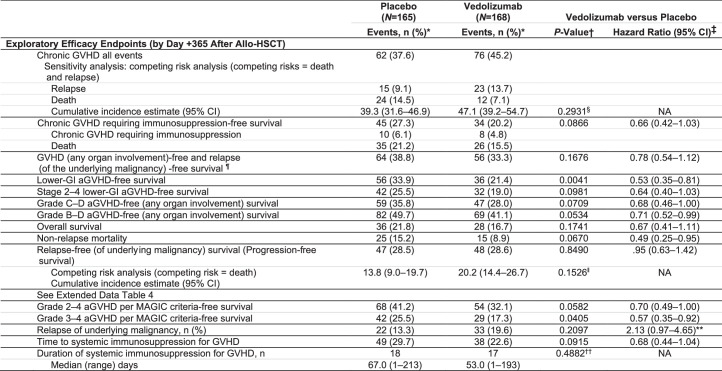
Results summary for main exploratory efficacy end points – by day +365

Results of the main exploratory study end points analyzed by day+365 after allo-HSCT. Analyses included all randomized patients who received ≥1 dose of study treatment and allo-HSCT. All statistical tests were two-sided.

* Number (%) of patients with an observed event/relapse/death (as applicable), whichever occurred first, from first study treatment dose (day −1) through day +365.

† Obtained via log-rank test unless otherwise stated.

‡ Obtained via Cox proportional hazards model with treatment group, stratified by randomization stratification factors: HLA match (7/8, 8/8), conditioning regimen (Myeloablative, Reduced Intensity), ATG (With, Without) unless otherwise stated.

§ Competing risk analysis with relapse and death as competing risks. *P*-value obtained by Gray’s test.

¶ GVHD and relapse-free survival was the time from the date of first study treatment dose (day-1) to GVHD event/death/relapse, where an event is defined as death or aGVHD grade 3–4 by modified Glucksberg criteria^9^ (Supplementary Table 2) or chronic GVHD requiring system immunosuppression or relapse.

‖Competing risk analysis with death as competing risk. *P*-value obtained by Gray’s test.

** Hazard ratio obtained via Cox proportional hazards model with treatment group adjusting for complete remission status and randomization stratification factors: HLA match or mismatch (8/8 or 7/8), Conditioning Regimen (Myeloablative, Reduced Intensity), and ATG (With, Without).

††*P*-value for comparison between vedolizumab and placebo was obtained using Wilcoxon rank-sum test. Allo-HSCT, allogenic hematopoietic stem cell transplantation; ATG, anti-thymocyte globulin; HLA, human leukocyte antigen; GVHD, graft-versus-host disease; MAGIC, Mount Sinai Acute GVHD International Consortium (Harris AC, et al. Biol Blood Marrow Transplant 2016;22:4-10).

**Extended Data Table 5:**
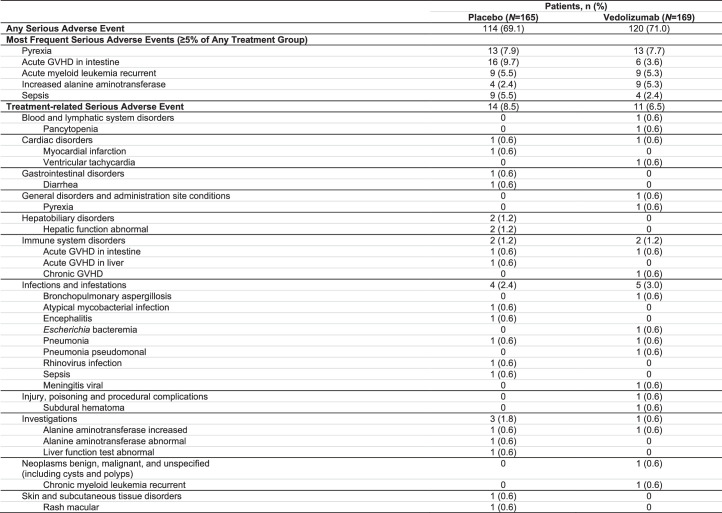
Serious adverse events

Safety analysis set included patients who received ≥1 dose of study treatment; includes adverse events (AEs) defined as any AE newly occurring or worsening after the first dose of study treatment to 18 weeks after last dose of study treatment.

Treatment-related AEs by system organ class and preferred term. MedDRA Dictionary (Version 24.0) was used for coding AEs.

GVHD, graft-versus-host disease.

**Extended Data Table 6:**
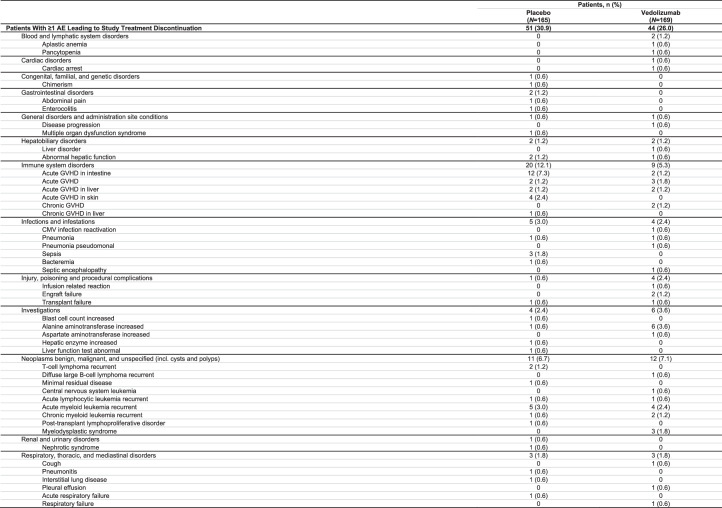
Adverse events leading to study treatment discontinuation

Safety analysis set included patients who received ≥1 dose of study treatment; includes adverse events (AEs) defined as any AE newly occurring or worsening after the first dose of study treatment to 18 weeks after last dose of study treatment.

AEs leading to study discontinuation by system organ class and preferred term. MedDRA Dictionary (version 24.0) was used for coding AEs.

CMV, cytomegalovirus; GVHD, graft-versus-host disease.

**Extended Data Table 7:**
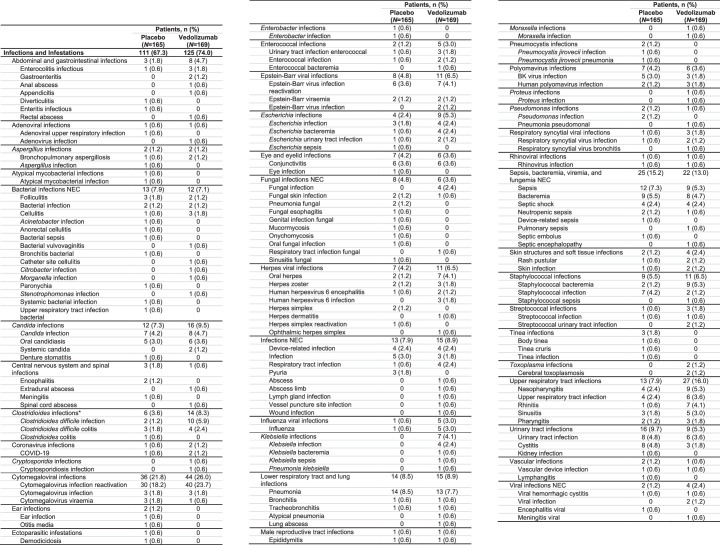
Adverse events of special interest: serious infections

Safety analysis set included patients who received ≥1 dose of study treatment; includes adverse events (AEs) defined as any AE newly occurring or worsening after the first dose of study treatment to 18 weeks after last dose of study treatment.

AEs leading to study discontinuation by system organ class and preferred term. MedDRA Dictionary (version 24.0) was used for coding AEs.

**Extended Data Table 8:**
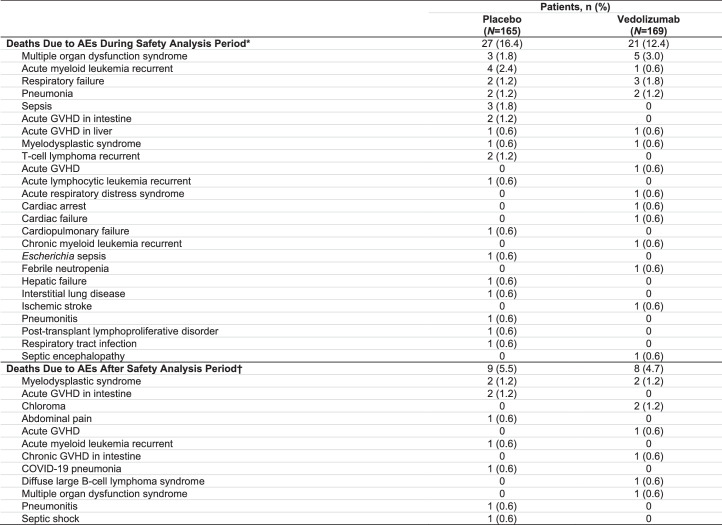
Deaths due to adverse events

Safety analysis set included patients who received ≥1 dose of study treatment. AEs defined as any AE newly occurring or worsening after the first dose of study treatment to 18 weeks after last dose of study treatment. MedDRA Dictionary (version 24.0) was used for coding AEs.

GVHD, graft-versus-host disease.

* Deaths due to adverse events (AEs) occurring during the safety analysis period from first dose of study treatment to 18 weeks after last dose of study treatment.

† Deaths due to AEs occurring in the time period after 18 weeks from the last dose of study treatment.

## Data Availability

The datasets, including the redacted study protocol, redacted statistical analysis plan and individual participants’ data supporting the results reported in this article, will be made available within 3 months from initial request to researchers who provide a methodologically sound proposal. The data will be provided after its de-identification, in compliance with applicable privacy laws, data protection, and requirements for consent and anonymization. Individual Participant Data from eligible studies will be shared with qualified researchers to address legitimate scientific objectives according to the criteria and process described at https://vivli.org/ourmember/takeda/. For approved requests, the researchers will be provided access to anonymized data (to respect patient privacy in line with applicable laws and regulations) and with information necessary to address the research objectives under the terms of a data sharing agreement. Individual Participant Data will be provided in a secure research environment following approval of a data sharing request, and under the terms of a data sharing agreement. There is no specified time frame for how long data will be shared.
