# Supplementary material for: Vedolizumab for the prevention of intestinal acute GVHD after allogeneic hematopoietic stem cell transplantation: a randomized phase 3 trial
Source: Nat Med. 2024 Jun 6;30(8):2277–87. doi: 10.1038/s41591-024-03016-4 (PMC11333288; doi:10.1038/s41591-024-03016-4)
Supplement: Supplementary file 1 — Supplementary Tables 1–10. [file 41591_2024_3016_MOESM1_ESM.pdf]

nature medicine

Supplementary information https://doi.org/10.1038/s41591-024-03016-4

Vedolizumab for the prevention of intestinal acute GVHD after allogeneic hematopoietic stem cell transplantation: a randomized phase 3 trial

In the format provided by the authors and unedited

SUPPLEMENTARY TABLES

TABLE OF CONTENTS

SUPPLEMENTARY TABLES 1

Table S1. aGVHD Clinical Stage.[1] 2
Table S2. aGVHD Grade (Modified Glucksberg).[1] 3
Table S3. Criteria for IBMTR Severity Index for aGVHD.[1] 4
Table S4. Criteria for MAGIC Severity Index for aGVHD.[1] 5
Table S5. ECOG Scale for Performance Status (For Patients Aged ≥18 Years).[1] 6
Table S6. Karnofsky and Lansky Performance Status Scales (For Patients Aged ≥16 and 12 to <16 Years, Respectively)[1] 7
Table S7. Incidence of aGVHD in lower-GI, upper-GI, skin, or liver by day +180 after allo-HSCT 8
Table S8. Sensitivity Analyses for the Secondary Endpoints. 9
Table S9. CMV Infections by Severity and by Use of ATG Prophylaxis. 10
Table S10. List of study centers 11

Table S1. aGVHD Clinical Stage.[1]

Stage	Skin*	Liver Bilirubin: SI Units (Standard Units)	Intestinal Tract† (Diarrhea/Day)	
			Aged ≥18 yr (or <18 yr and ≥50 kg)	Aged <18 yr and <50 kg
**1**	Maculopapular rash <25% of BSA	34–50 µmol/L (2–3 mg/dL)	>500 mL diarrhea/day	10–19.9 mL/kg/d
**2**	Maculopapular rash 25%–50% of BSA	51–102 µmol/L (3.1–6 mg/dL)	>1000 mL diarrhea/day	20–30 mL/kg/d
**3**	Rash >50% of BSA	103–255 µmol/L (6.1–1.5 mg/dL)	>1500 mL diarrhea/day	>30 mL/kg/d
**4**	Generalized erythroderma with bullous formation	>255 µmol/L (>15 mg/dL)	Severe abdominal pain, with or without ileus	Severe abdominal pain, with or without ileus

* Use the "Rule of Nines" or burn chart to determine the extent of the rash.

† Staging of intestinal tract should be assessed based on patient's age and weight at the time of assessment.

aGVHD, acute graft-versus-host disease; BSA, body surface area.

1. Przepiorka D, Weisdorf D, Martin P, et al. 1994 Consensus Conference on Acute GVHD Grading. Bone Marrow Transplant 1995;15:825-8

Table S2. aGVHD Grade (Modified Glucksberg).[1]

Grade	Skin	Liver	Intestinal Tract
**I**	Stage 1–2	None	None
**II**	Stage 3 *or* →	Stage 1 *or* →	Stage 1
**III**	–	Stage 2–3 *or* →	Stage 2-4
**IV**	Stage 4 *or* →	Stage 4	–

aGVHD, acute graft-versus-host disease.

1. Przepiorka D, Weisdorf D, Martin P, et al. 1994 Consensus Conference on Acute GVHD Grading. Bone Marrow Transplant 1995;15:825-8.

Table S3. Criteria for IBMTR Severity Index for aGVHD.[1]

	Skin	Liver	Intestinal Tract
**Index**	**Stage (Max)**	**Stage (Max)**	**Stage (Max)**
**A**	Stage 1	Stage 0	Stage 0
**B**	Stage 2 *or*	Stage 1–2 *or*	Stage 1–2
**C**	Stage 3 *or*	Stage 3 *or*	Stage 3
**D**	Stage 4 *or*	Stage 4 *or*	Stage 4

aGVHD, acute graft-versus-host disease; IBMTR, International Bone Marrow Transplant Registry.

1. Rowlings PA, Przepiorka D, Klein JP, et al. IBMTR Severity Index for grading acute graft-versus-host disease: retrospective comparison with Glucksberg grade. Br J Haematol 1997;97:855-64.

Table S4. Criteria for MAGIC Severity Index for aGVHD.[1]

Stage*	Skin (Active Erythema Only)	Liver (Bilirubin)	Upper GI	Intestinal Tract (Stool Output/Day)	
				Aged ≥ 18 yr (or <18 yr and ≥ 50 kg)	Aged < 18 yr and <50 kg
**0**	No active (erythematous) GVHD rash	<2 mg/dL	No or intermittent nausea, vomiting, or anorexia	<500 mL/day or <3 episodes/day	<10 mL/kg/day or <4 episodes/day
**1**	Maculopapular rash <25% of body surface	2–3 mg/dL	Persistent nausea, vomiting, or anorexia	500–999 mL/day or 3–4 episodes/day	10–19.9 mL/kg/day or 4–6 episodes/day
**2**	Maculopapular rash 25–50% of body surface	3.1–6 mg/dL	–	1000–1500 mL/day or 5–7 episodes/day	20–30 mL/kg/day or 7–10 episodes/day
**3**	Maculopapular rash >50% of body surface	6.1–15 mg/dL	–	>1500 mL/day or >7 episodes/day	>30 mL/kg/day or >10 episodes/day
**4**	Generalized erythroderma (>50% BSA) + bullous formation and desquamation>5% body surface	>15 mg/dL	–	Severe abdominal pain, with or without ileus, or grossly bloody stool (regardless of stool volume)	Severe abdominal pain, with or without ileus, or grossly bloody stool (regardless of stool volume)

* Overall clinical grade (based on most severe target organ involvement): Grade 0: No Stage 1–4 of any organ; Grade I: Stage 1–2 skin without liver, upper GI or lower-GI involvement; Grade II: Stage 3 rash and/or Stage 1 liver and/or Stage 1 upper GI and/or Stage 1 lower GI; Grade III: Stage 2–3 liver and/or Stage 2–3 lower GI, with Stage 0–3 skin and/or Stage 0–1 upper GI; Grade IV: Stage 4 skin, liver or lower-GI involvement with Stage 0–1 upper GI.

† Staging of intestinal tract should be assessed based on patient's age and weight at the time of assessment.

aGVHD, acute graft-versus-host disease; BSA, body surface area; GI gastrointestinal; GVHD, graft-versus-host disease; MAGIC, Mount Sinai Acute GVHD International Consortium.

1. Harris AC, Young R, Devine S, et al. International, Multicenter Standardization of Acute Graft-versus-Host Disease Clinical Data Collection: A Report from the Mount Sinai Acute GVHD International Consortium. Biol Blood Marrow Transplant 2016;22:4-10.

Table S5. ECOG Scale for Performance Status (For Patients Aged ≥18 Years).[1]

Grade	Description
**0**	Normal activity. Fully active, able to carry on all pre-disease performance without restriction.
**1**	Symptoms but ambulatory. Restricted in physically strenuous activity but ambulatory and able to carry out work of a light or sedentary nature (e.g., light housework, office work).
**2**	In bed <50% of the time. Ambulatory and capable of all self-care but unable to carry out any work activities. Up and about >50% of waking hours.
**3**	In bed >50% of the time. Capable of only limited self-care, confined to bed or chair >50% of waking hours.
**4**	100% bedridden. Completely disabled. Cannot carry on any self-care. Totally confined to bed or chair.
**5**	Dead.

ECOG, Eastern Cooperative Oncology Group.

1. Oken MM, Creech RH, Tormey DC, et al. Toxicity and response criteria of the Eastern Cooperative Oncology Group. Am J Clin Oncol 1982;5:649-55.

Table S6. Karnofsky and Lansky Performance Status Scales
(For Patients Aged ≥16 and 12 to <16 Years, Respectively)[1]

Karnofsky Scale (Recipient Age ≥16 Years)		Lansky Scale (Recipient Age 12 to <16 Years)	
**Score**	**Description**	**Score**	**Description**
Able to carry on normal activity; no special care is needed		***Able to carry on normal activity; no special care is needed***	
100	Normal, no complaints, no evidence of disease	100	Fully active
90	Able to carry on normal activity	90	Minor restriction in physically strenuous play
80	Normal activity with effort	80	Restricted in strenuous play, tires more easily, otherwise active
Unable to work, able to live at home, cares for most personal needs, a varying amount of assistance is needed		***Mild to moderate restriction***	
70	Cares for self, unable to carry on normal activity or to do active work	70	Both greater restrictions of, and less time spent in active play
60	Requires occasional assistance but is able to care for most needs	60	Ambulatory up to 50% of time, limited active play with assistance/supervision
50	Requires considerable assistance and frequent medical care	50	Considerable assistance required for any active play, fully able to engage in quiet play
	Unable to care for self, requires equivalent of institutional or hospital care, disease may be progressing rapidly		Moderate to severe restriction
40	Disabled, requires special care and assistance	40	Able to initiate quiet activities
30	Severely disabled, hospitalization indicated, although death not imminent	30	Needs considerable assistance for quiet activity
20	Very sick, hospitalization necessary	20	Limited to very passive activity initiated by others (e.g., TV)
10	Moribund, fatal process progressing rapidly	10	Completely disabled, not even passive play
0	Dead		

1. Center for International Blood & Marrow Transplant Research CIBMTR.org Center for International Blood & Marrow Transplant Research. Forms Instruction Manual. Appendix L: Karnofsky/Lansky Performance Status pg 2064, 2009 (https://cdn.manula.com/user/3235/3235_3360_3509_en_1418684645.pdf?v=20230828103227).

Table S7. Incidence of aGVHD in lower-GI, upper-GI, skin, or liver by day +180 after allo-HSCT

	**Placebo (N=165)** n (%)	**Vedolizumab (N=168)** n (%)	**Adjusted treatment difference** (95% CI)	***P*-value by stratified CMH**
**Overall incidence of lower-GI aGVHD** (lower-GI aGVHD alone plus combined upper- and lower-GI aGVHD)	31 (18.8)	12 (7.1)	-11.7 (-18.9 to -4.5)	0.0016
**Overall incidence of upper-GI aGVHD** (upper-GI aGVHD alone plus combined upper- and lower-GI aGVHD)	19 (11.5)	15 (8.9)	-2.6 (-9.0 to 3.8)	0.4366
**Overall incidence of skin aGVHD**	76 (46.1)	77 (45.8)	-0.2 (-10.8 to 10.5)	0.9713
**Overall incidence of liver aGVHD**	8 (4.9)	12 (7.1)	2.1 (-2.8 to 7.0)	0.4038

For GI aGVHD location at day+180 after allo-HSCT, study investigators were asked to select an option for location of GI aGVHD as ‘upper intestinal, lower intestinal or both’. *P*-value for difference between treatment groups was obtained by two-sided CMH test, adjusting for randomization stratification factors: HLA match or mismatch (8/8 or 7/8), conditioning regimen (myeloablative or reduced intensity conditioning), and treatment with or without ATG.

aGVHD, acute graft-versus-host disease; allo-HSCT, allogenic hematopoietic stem cell transplantation; ATG, anti-thymocyte globulin; CMH, Cochran–Mantel–Haenszel test; GI, gastrointestinal.

Table S8. Sensitivity Analyses for the Secondary Endpoints.

	Placebo (N=165)		Vedolizumab (N=168)			
	**Event, n (%)***	**KM Estimated Survival, %**	**Event, n (%)***	**KM Estimated Survival, %**	***P*-value†**	**Hazard Ratio (95% CI)‡**
**1st** Lower-GI aGVHD-free and relapse-free survival by day +180						
Primary analysis §	56 (33.9)	65.4	35 (20.8)	78.9	0.0043	0.56 (0.37–0.86)
Sensitivity 1: using day +187 window ¶	57 (34.5)	64.7	36 (21.4)	78.3	0.0045	0.57 (0.37–0.86)
Sensitivity 2: by stratified log-rank test ‖	56 (33.9)	65.4	35 (20.8)	78.9	0.0067	0.56 (0.37–0.86)
Sensitivity 3: by competing risk analysis (competing risk is non-relapse mortality) **	44 (26.7)	27.2 (Cumulative incidence, %)	29 (17.3)	17.5 (Cumulative incidence, %)	0.0260	NA
Sensitivity 4: with corrected stratification information ††	56 (33.9)	65.4	35 (20.8)	78.9	0.0043	0.56 (0.37–0.86)
Sensitivity 5: with clinical stage 0 and stage unknown removed ‡‡	52 (31.5)	67.8	34 (20.2)	79.5	0.0130	0.59 (0.38–0.91)
**2nd** Grade C-D aGVHD (Based on IBMTR index for Any Organ Involvement) Free Survival by day +180						
Primary analysis §§	52 (31.5)	67.7	35 (20.8)	78.9	0.0204	0.59 (0.39–0.91)
Sensitivity 1: using day +187 window ¶	53 (32.1)	67.1	35 (20.8)	78.9	0.0154	0.58 (0.38–0.90)
Sensitivity 2: by stratified log-rank test ‖	52 (31.5)	67.7	35 (20.8)	78.9	0.0165	0.59 (0.39–0.91)
Sensitivity 3: by competing risk analysis (competing risk is death and relapse) **	35 (21.2)	21.6 (Cumulative incidence, %)	22 (13.1)	13.2 (Cumulative incidence, %)	0.0446	NA
Sensitivity 4: with corrected stratification information ††	52 (31.5)	67.7	35 (20.8)	78.9	0.0204	0.60 (0.39–0.93)
**3rd** Non-relapse mortality by day +180						
Primary analysis ¶¶	19 (11.5)	88.0	10 (6.0)	93.9	0.0668	0.48 (0.22–1.04)
Sensitivity 1: using day +187 window ¶	19 (11.5)	88.0	10 (6.0)	93.9	0.0668	0.48 (0.22–1.04)
Sensitivity 2: by stratified log-rank test ‖	19 (11.5)	88.0	10 (6.0)	93.9	0.0572	0.48 (0.22–1.04)
Sensitivity 3: by competing risk analysis (competing risk is relapse) **	19 (11.5)	11.8 (Cumulative incidence, %)	10 (6.0)	6.0 (Cumulative incidence, %)	0.0698	NA
Sensitivity 4: with corrected stratification information ††	19 (11.5)	88.0	10 (6.0)	93.9	0.0668	0.49 (0.23–1.05)
**4th** Overall survival by day +180						
Primary analysis ‖‖	25 (15.2)	84.4	17 (10.1)	89.7	0.1458	0.63 (0.34–1.17)
Sensitivity 1: using day +187 window ¶	26 (15.8)	83.7	17 (10.1)	89.7	0.1098	0.60 (0.33–1.11)
Sensitivity 2: by stratified log-rank test ‖	25 (15.2)	84.4	17 (10.1)	89.7	0.1367	0.63 (0.34–1.17)
Sensitivity 3: by competing risk analysis **	NA	NA	NA	NA	NA	NA
Sensitivity 4: with corrected stratification information ††	25 (15.2)	84.4	17 (10.1)	89.7	0.1458	0.64 (0.34–1.18)
**5th** Grade B-D aGVHD Free (Based on IBMTR Severity Index for Any Organ Involvement) Survival by day +180						
Primary analysis ***	77 (46.7)	52.3	56 (33.3)	66.4	0.0105	0.64 (0.46–0.91)
Sensitivity 1: Using day +187 window ¶	78 (47.3)	51.7	57 (33.9)	65.7	0.0103	0.65 (0.46–0.91)
Sensitivity 2: by stratified log-rank test ‖	77 (46.7)	52.3	56 (33.3)	66.4	0.0120	0.64 (0.46–0.91)
Sensitivity 3: by competing risk analysis (competing risk is death and relapse) **	64 (38.8)	39.5 (Cumulative incidence, %)	47 (28.0)	28.2 (Cumulative incidence, %)	0.0321	NA
Sensitivity 4: with corrected stratification information ††	77 (46.7)	52.3	56 (33.3)	66.4	0.0105	0.64 (0.45–0.91)

Prespecified sensitivity analyses for the secondary efficacy endpoints: day +187 window, stratified log-rank test, competing risk analysis, and corrected stratification information. Analyses included all randomized patients who received ≥1 dose of study treatment and received allo-HSCT. All statistical tests were two-sided.

* Number (%) of patients with an observed event/relapse/death (as applicable), whichever occurred first, from first study treatment dose (day −1) through day +180 or day +365. Full analysis set includes all randomized patients who received ≥1 dose of study treatment and received allogeneic HSCT.† *P*-value for the comparison between vedolizumab and placebo was obtained by log-rank test, unless otherwise stated. ‡ Hazard ratio was obtained via Cox proportional-hazards model with treatment group, stratified by randomization stratification factors: HLA match (7/8, 8/8), Conditioning Regimen (Myeloablative, Reduced Intensity), ATG (With, Without), unless otherwise stated. § Lower-GI aGVHD event, relapse, or death are defined as events in the analysis. ¶ Sensitivity 1 is an analysis of events occurring within a 7-day window at day 187 after allo-HSCT. ‖ Stratified log-rank test in Sensitivity 2 was used to compare treatment groups stratified by randomization stratification factors: HLA Match (7/8, 8/8), Conditioning Regimen (Myeloablative, Reduced Intensity), ATG (With, Without). ** In Sensitivity 3, *P*-value for comparison of vedolizumab with placebo was obtained by Gray's test. †† Sensitivity 4 analysis used corrected information for randomization strata: HLA Match (7/8, 8/8), Conditioning Regimen (Myeloablative, Reduced Intensity), ATG (With, Without). ‡‡ Excluding Intestinal aGVHD Clinical Stage 0 and Unknown Clinical Stage from the Definition of an Intestinal aGVHD Event. §§ Grade C–D aGVHD event per IBMTR Severity Index or death are defined as events in the analysis. ¶¶ Deaths without relapse are defined as events in the analysis.‖‖ All-cause deaths are defined as events in the analysis.*** Grade B–D aGVHD event per IBMTR Severity Index or death are defined as events in the analysis.

aGVHD, acute graft-versus-host disease; IBMTR, International Bone Marrow Transplant Registry. aGVHD, acute graft-versus-host disease; ATG, anti-thymocyte globulin; HLA, human leukocyte antigen; HSCT, hematopoietic stem cell transplantation.

Table S9. CMV Infections by Severity and by Use of ATG Prophylaxis.

CMV Infections by Severity and ATG Use (SAF*)	Placebo (n=165)		Vedolizumab (n=168)	
	Events, n	Patients, n (%)	Events, n	Patients, n (%)
**With ATG**				
CMV infections	30	21 (12.7)	40	28 (16.6)
Grade 1	7	3 (1.8)	9	5 (3.0)
Grade 2	17	12 (7.3)	20	16 (9.5)
Grade 3	5	5 (3.0)	11	7 (4.1)
Grade 4	1	1 (0.6)	0	0
Grade 5	0	0	0	0
Grade ≥3	6	6 (3.6)	11	7 (4.1)
Serious CMV infections	4	3 (1.8)	9	7 (4.1)
**Without ATG**				
CMV infections	22	16 (9.7)	21	17 (10.1)
Grade 1	5	3 (1.8)	6	4 (2.4)
Grade 2	12	10 (6.1)	14	12 (7.1)
Grade 3	5	3 (1.8)	1	1 (0.6)
Grade 4	0	0	0	0
Grade 5	0	0	0	0
Grade ≥3	5	3 (1.8)	1	1 (0.6)
Serious CMV infections	0	0	1	1 (0.6)

* (SAF) Safety analysis set included patients who received ≥1 dose of study treatment. Table includes adverse events (AEs) defined as any AE newly occurring or worsening after the first dose of study treatment to 18 weeks after last dose of study treatment.

aGVHD, acute graft-versus-host disease; ATG, anti-thymocyte globulin; CMV, cytomegalovirus.

Table S10. List of study sites

Country	Site name
**Canada**	CancerCare Manitoba, Winnipeg, MB
**USA**	David Geffen School of Medicine at University of California Los Angeles, Los Angeles, CA
	University of Alabama at Birmingham Comprehensive Cancer Center, Birmingham, AL
	Marlene and Stewart Greenebaum Cancer Center, Baltimore, MD
	Loyola University Medical Center, Maywood, IL
	Barbara Ann Karmanos Cancer Institute, Detroit, MI
	Indiana University Melvin and Bren Simon Cancer Center, Indianapolis, IN
	University of North Carolina Hospitals, Chapel Hill, NC
	Beth Israel Deaconess Medical Center, Boston, MA
	Emory University - Winship Cancer Institute, Atlanta, GA
	Massachusetts General Hospital, Boston, MA
	Columbia University Medical Center - The Columbia Center for Translational Immunology, New York, NY
	Oregon Health and Science University, Portland, OR
	Roswell Park Cancer Institute, Buffalo, NY
	University of Virginia Health System, Charlottesville, VA
	University of Kentucky Chandler Medical Center, Lexington, KY
	Augusta University Georgia Cancer Center, August, GA
	Dana Farber Cancer Institute, Boston, MA
	Mayo Clinic – Rochester, Rochester, MN
**Argentina**	Fundación Favaloro Hospital Universitario, Ciudad Autonoma de Buenos Aires, Buenos Aires
	Hospital Privado Centro Médico de Córdoba, Córdoba
	Ciudad Autonoma de Buenos Aires, Buenos Aires
**Brazil**	Instituto Brasileiro de Controle do Câncer - São Camilo Oncolgia, São Paulo
	Hospital Santa Marcelina, São Paulo
	Hospital de Câncer de Barretos, Barretos, São Paulo
	Hospital Universitário Walter Cantídio - Universidade Federal do Ceará
**Belgium**	Universitair Ziekenhuis Leuven, Leuven, Flemish Brabant
	Ziekenhuisnetwerk Stuivenberg, Antwerpen
**France**	Hôpital Saint Louis, Paris Cedex 10, Ile-De-France
	Centre Hospitalier Universitaire de Limoges, Limoges Cedex, Limousin, Lorraine
	Hôpital Saint-Antoine, Paris Cedex 12, Ile-De-France
	Hôpital Pontchaillou, Rennes Cedex 9, Bretagne
	Hôpital Necker-Enfants Malades, Paris, Ile-De-France
	Centre Hospitalier Universitaire Amiens-Picardie, Amiens, Picardie
	Centre Hospitalier Universitaire Nantes - Hotel Dieu, Nantes Cedex 1, Pays De La Loire
**Germany**	Diakonie-Klinikum Stuttgart, Stuttgart, Baden-Wuerttemberg
	Universitätsklinikum Halle, Halle, Sachsen-Anhalt
	Universitätsmedizin Mannheim, Mannheim, Baden-Wuerttemberg
	Universitaetsklinikum Schleswig-Holstein - Campus Kiel, Kiel, Schleswig-Holstein
	Universitätsklinikum Carl Gustav Carus Dresden, Dresden, Sachsen
**Italy**	Azienda Grande Ospedale Metropolitano Bianchi Melacrino Morelli, Reggio Calabria, CALABRIA
	Policlinico Universitario di Catania, Catania
	Istituto Clinico Humanitas Humanitas Cancer Center, Milano
	Azienda Ospedaliera Cardinale G. Panico, Tricase, Lecce
	Ospedale Mazzoni, Ascoli Piceno, Marche
	Presidio Ospedaliero di Pescara, Pescara
	Ospedale dell'Angelo, Mestre, Venezia
**Norway**	Oslo University Hospital – Rikshospitalet, Oslo
**Portugal**	Instituto Português de Oncologia do Porto Francisco Gentil, Porto
**Spain**	Hospital Universitario Virgen de la Arrixaca, Murcia
	Hospital Clínico Universitario de Valencia, Valencia
	Hospital Universitario Virgen del Rocio, Sevilla
	Hospital General Universitario Morales Meseguer, Murcia
	Hospital Universitario de Salamanca, Salamanca
	Hospital Universitario de Gran Canaria Doctor Negrin, Las Palmas de GC
**Sweden**	Karolinska Universitetssjukhuset, Stockholm
	Skånes Universitetssjukhus i Lund, Lund, Skane
**Switzerland**	Universitätsspital Zürich, Zurich
	Universitätsspital Basel, Basel
**UK**	Sheffield Teaching Hospitals NHS Foundation Trust, Sheffield
	Cardiff and Vale University Health Board, Cardiff
	Barts Health NHS Trust, London
**Austria**	Ordensklinikum Linz Elisabethinen, Linz, Upper Austria
**Greece**	University Regional General Hospital of Patras, Patras, Peloponnese
**Hungary**	Debreceni Egyetem Klinikai Központ, Debrecen, Hajdu-Bihar
**Poland**	Uniwersyteckie Centrum Kliniczne, Gdańsk, Pomorskie
**Israel**	Rambam Health Care Campus - Rambam Medical Center, Haifa
	Hadassah Medical Center, Jerusalem
**Romania**	Institutul Clinic Fundeni, Bucurest
**Russia**	Sverdlovsk Regional Clinical Hospital #1, Ekaterinburg, Sverdlovsk
	National Research Center for Hematology, Moscow
**Australia**	Royal Brisbane and Women's Hospital, Herston
	The Royal Melbourne Hospital, Parkville, Victoria
	Fiona Stanley Hospital, Murdoch, WA
	Saint Vincent's Hospital Sydney, Darlinghurst, NSW
	Austin Health, Heidelberg, Victoria
**Japan**	Hokkaido University Hospital, Sapporo-shi, Hokkaido
	Hiroshima Red Cross Hospital and Atomic-bomb Survivors Hospital, Hiroshima-city, Hiroshima
	Osaka City University Hospital, Osaka
	Okayama University Hospital, Okayama-city, Okayama
	Kyushu University Hospital, Fukuoka-shi, Fukuoka
	Japanese Red Cross Aichi Medical Center Nagoya Daiichi Hospital, Nagoya, Aichi
	Tokyo Metropolitan Cancer and Infectious diseases Center Komagome Hospital, Bunkyo-Ku, Tokyo
	Jichi Medical University Hospital, Shimotsuke-city, Tochigi
	Shizuoka Cancer Center, Sunto-gun, Shizuoka
**Republic of Korea**	Seoul National University Hospital, Seoul, Gyeonggi-do
	Chungnam National University Hospital, Daejeon
	Kyungpook National University Hospital, Daegu, Gyeongsangbuk-do
	Seoul National University Bundang Hospital, Seongnam-si, Gyeonggi-do
	Pusan National University Hospital, Busan, Gyeongsangnam-do
	Severance Hospital, Seoul
	Daegu Catholic University Medical Center, Daegu
	Keimyung University Dongsan Hospital, Daegu, Gyeongsangbuk-do
**Singapore**	Raffles Hospital, Singapore
	National University Hospital, Singapore
