# Supplementary material for: Vedolizumab for the prevention of intestinal acute GVHD after allogeneic hematopoietic stem cell transplantation: a randomized phase 3 trial
Source: Nat Med. 2024 Jun 6;30(8):2277–87. doi: 10.1038/s41591-024-03016-4 (PMC11333288; doi:10.1038/s41591-024-03016-4)
Supplement: Supplementary file 2 — Reporting Summary [file 41591_2024_3016_MOESM2_ESM.pdf]

nature portfolio

Corresponding author(s): Yi-Bin Chen

Last updated by author(s): Apr 22, 2024

Reporting Summary

Nature Portfolio wishes to improve the reproducibility of the work that we publish. This form provides structure for consistency and transparency in reporting. For further information on Nature Portfolio policies, see our Editorial Policies and the Editorial Policy Checklist.

Statistics

For all statistical analyses, confirm that the following items are present in the figure legend, table legend, main text, or Methods section.

n/a	Confirmed	
☐	☒	The exact sample size (n) for each experimental group/condition, given as a discrete number and unit of measurement
☐	☒	A statement on whether measurements were taken from distinct samples or whether the same sample was measured repeatedly
☐	☒	The statistical test(s) used AND whether they are one- or two-sided<br>*Only common tests should be described solely by name; describe more complex techniques in the Methods section.*
☐	☒	A description of all covariates tested
☐	☒	A description of any assumptions or corrections, such as tests of normality and adjustment for multiple comparisons
☐	☒	A full description of the statistical parameters including central tendency (e.g. means) or other basic estimates (e.g. regression coefficient) AND variation (e.g. standard deviation) or associated estimates of uncertainty (e.g. confidence intervals)
☒	☐	For null hypothesis testing, the test statistic (e.g. F, t, r) with confidence intervals, effect sizes, degrees of freedom and P value noted<br>*Give P values as exact values whenever suitable.*
☒	☐	For Bayesian analysis, information on the choice of priors and Markov chain Monte Carlo settings
☐	☒	For hierarchical and complex designs, identification of the appropriate level for tests and full reporting of outcomes
☒	☐	Estimates of effect sizes (e.g. Cohen's d, Pearson's r), indicating how they were calculated

Our web collection on statistics for biologists contains articles on many of the points above.

Software and code

Policy information about availability of computer code

Data collection	No software used
Data analysis	SAS Version 9.4, or higher

For manuscripts utilizing custom algorithms or software that are central to the research but not yet described in published literature, software must be made available to editors and reviewers. We strongly encourage code deposition in a community repository (e.g. GitHub). See the Nature Portfolio guidelines for submitting code & software for further information.

Data

Policy information about availability of data

All manuscripts must include a data availability statement. This statement should provide the following information, where applicable:

- Accession codes, unique identifiers, or web links for publicly available datasets
- A description of any restrictions on data availability
- For clinical datasets or third party data, please ensure that the statement adheres to our policy

The datasets, including the redacted study protocol, redacted statistical analysis plan and individual participants' data supporting the results reported in this article, will be made available within 3 months from initial request to researchers who provide a methodologically sound proposal. The data will be provided after its de-identification, in compliance with applicable privacy laws, data protection, and requirements for consent and anonymization. Individual Participant Data (IPD) from eligible studies will be shared with qualified researchers to address legitimate scientific objectives according to the criteria and process described on https://

vivli.org/ourmember/takeda/. For approved requests, the researchers will be provided access to anonymized data (to respect patient privacy in line with applicable laws and regulations) and with information necessary to address the research objectives under the terms of a data sharing agreement. IPDs will be provided in a secure research environment following approval of a data sharing request, and under the terms of a data sharing agreement. There is no specified time frame for how long data will be shared.

Research involving human participants, their data, or biological material

Policy information about studies with human participants or human data. See also policy information about sex, gender (identity/presentation), and sexual orientation and race, ethnicity and racism.

Reporting on sex and gender	Reporting of patient demographic data included counts of the number of patients of each biological sex in the study sample analyzed. These data were reported by screening personnel at patient screening study visit (within 30 days of first dose of study treatment). Pre-specified sub-group analyses of the primary and secondary endpoints included sub-group analyses by biological sex.
Reporting on race, ethnicity, or other socially relevant groupings	Reporting of patient demographic data included counts of the number of patients in each race and ethnicity category in the study sample analyzed. These data were reported by screening personnel at patient screening study visit (within 30 days of first dose of study treatment). Pre-specified sub-group analyses of the primary endpoints included sub-group analyses by race and sex.
Population characteristics	Median age was 55.0 years (range, 16–74 years; 1 aged <18 years); 62.8% were male. The most frequent underlying malignancies were acute myeloid leukemia (AML), myelodysplastic syndrome (MDS) and acute lymphoid leukemia (ALL).
Recruitment	Patients were recruited at hospital centers participating in the study, only patients who provided written informed consent and met the eligibility criteria pre-specified in the study protocol and approved by the IRB of participating centers were eligible for study enrollment.
Ethics oversight	Local institutional review boards of all participating centers approved the study protocol (see Supplementary Table 10 for list of study centers)

Note that full information on the approval of the study protocol must also be provided in the manuscript.

Field-specific reporting

Please select the one below that is the best fit for your research. If you are not sure, read the appropriate sections before making your selection.

☒ Life sciences ☐ Behavioural & social sciences ☐ Ecological, evolutionary & environmental sciences

For a reference copy of the document with all sections, see nature.com/documents/nr-reporting-summary-flat.pdf

Life sciences study design

All studies must disclose on these points even when the disclosure is negative.

Sample size	A sample size of approximately 558 patients, (assuming an event rate of 34.1% for the placebo group and 21.8% for the vedolizumab group and 10% loss to follow-up for both groups) was planned to generate 148 primary efficacy events to detect a target HR of 0.59 between vedolizumab and placebo with 90% power for the primary endpoint based on a two-sided log-rank test at a significance level of 0.05.
Data exclusions	Following the intent-to-treat principle, all randomized patients receiving ≥1 dose of study treatment and who underwent allo-HSCT were analyzed for efficacy endpoints. All patients who received ≥1 dose of the study treatment were analyzed for safety endpoints.
Replication	Secondary endpoints employed different sets of measures for clinical grading of the extent and severity of acute GVHD (IBMTR grading) to those used for the primary endpoint, as well as alternative outcome measures of treatment efficacy (relapse of the primary malignancy, relapse-free mortality and aGVHD of any organ), as methods of verifying findings from primary outcomes data. Pre-specified sensitivity analyses for the primary and secondary study endpoints were also performed. Results were consistent for sensitivity analyses of the primary endpoint, including events occurring within a 7-day timeframe at day +187 after allo-HSCT, stratified log-rank tests by randomization stratification factors, analysis with corrected stratification information, competing risk analysis and an analysis excluding aGVHD events graded stage 0 or unknown. In prespecified subgroup analyses, HRs consistently favored vedolizumab over placebo regardless of HLA match, conditioning regimen intensity, use of ATG, or stem-cell source (bone marrow or peripheral blood).
Randomization	Patients were randomized 1:1 to treatment groups using an interactive response technology system
Blinding	Double-blind

Reporting for specific materials, systems and methods

We require information from authors about some types of materials, experimental systems and methods used in many studies. Here, indicate whether each material, system or method listed is relevant to your study. If you are not sure if a list item applies to your research, read the appropriate section before selecting a response.

Materials & experimental systems

n/a	Involved in the study
☒	☐ Antibodies
☒	☐ Eukaryotic cell lines
☒	☐ Palaeontology and archaeology
☒	☐ Animals and other organisms
☐	☒ Clinical data
☒	☐ Dual use research of concern
☒	☐ Plants

Methods

n/a	Involved in the study
☒	☐ ChIP-seq
☒	☐ Flow cytometry
☒	☐ MRI-based neuroimaging

Clinical data

Policy information about clinical studies

All manuscripts should comply with the ICMJE guidelines for publication of clinical research and a completed CONSORT checklist must be included with all submissions.

Clinical trial registration	NCT03657160
Study protocol	Data sharing statement provided with the manuscript states that the redacted protocol will be made available upon request.
Data collection	Study enrollment ran from February 06 2019 through May 09 2022, at 95 hospital transplant centers: 19 in North America, 7 in South America, 45 in Europe and 24 in Asia/Australia see Supplementary Table 10 for list of study centers. Study completion date was May, 2022.
Outcomes	Primary and secondary outcomes were pre-defined in the study protocol before study commencement and were assessed in a prospective, randomized, placebo-controlled, double-blind trial of patients on an intent to treat basis. All study participants provided their written informed consent. The primary outcome of lower-GI aGVHD-free survival and secondary outcomes of lower-GI aGVHD-free and relapse of the underlying malignancy-free survival; IBMTR Grade C–D aGVHD of any organ-free survival; non-relapse mortality; overall survival; and IBMTR Grade B–D aGVHD of any organ-free survival were assessed by the study investigators based on their assessment of patient clinical data.

Plants

Seed stocks	n/a
Novel plant genotypes	n/a
Authentication	n/a
